# Lactylation‐Driven HECTD2 Limits the Response of Hepatocellular Carcinoma to Lenvatinib

**DOI:** 10.1002/advs.202412559

**Published:** 2025-02-20

**Authors:** Runyu Dong, Yao Fei, Yiren He, Peng Gao, Bo Zhang, Menglin Zhu, Zhixiong Wang, Longfei Wu, Shuai Wu, Xiaoming Wang, Juan Cai, Zhiqiang Chen, Xueliang Zuo

**Affiliations:** ^1^ Department of General Surgery The First Affiliated Hospital of USTC Division of Life Sciences and Medicine University of Science and Technology of China Hefei 230001 China; ^2^ Department of Gastrointestinal Surgery The First Affiliated Hospital Yijishan Hospital of Wannan Medical College Wuhu 241000 China; ^3^ Department of Oncology The First Affiliated Hospital Yijishan Hospital of Wannan Medical College Wuhu 241000 China; ^4^ Department of Hepatobiliary Surgery The First Affiliated Hospital Yijishan Hospital of Wannan Medical College Wuhu 241000 China; ^5^ Anhui Province Key Laboratory of Non‐coding RNA Basic and Clinical Transformation Wannan Medical College Wuhu 241000 China; ^6^ Department of Oncology The First Affiliated Hospital of USTC Division of Life Sciences and Medicine University of Science and Technology of China Hefei 230001 China; ^7^ Hepatobiliary Center The First Affiliated Hospital of Nanjing Medical University Key Laboratory of Liver Transplantation Chinese Academy of Medical Sciences NHC Key Laboratory of Hepatobiliary Cancers Nanjing 210000 China

**Keywords:** HECTD2, histone lactylation, nanoparticles, oxidative stress, ubiquitination

## Abstract

Drug resistance remains a major hurdle for the therapeutic efficacy of lenvatinib in hepatocellular carcinoma (HCC). However, the underlying mechanisms remain largely undetermined. Unbiased proteomic screening is performed to identify the potential regulators of lenvatinib resistance in HCC. Patient‐derived organoids, patient‐derived xenograft mouse models, and DEN/CCl_4_ induced HCC models are constructed to evaluate the effects of HECTD2 both in vitro and in vivo. HECTD2 is found to be highly expressed in lenvatinib‐resistant HCC cell lines, patient tissues, and patient‐derived organoids and xenografts. In vitro and in vivo experiments demonstrated that overexpression of HECTD2 limits the response of HCC to lenvatinib treatment. Mechanistically, HECTD2 functions as an E3 ubiquitin ligase of KEAP1, which contributes to the degradation of KEAP1 protein. Subsequently, the KEAP1/NRF2 signaling pathway initiates the antioxidative response of HCC cells. Lactylation of histone 3 on lysine residue 18 facilitates the transcription of HECTD2. Notably, a PLGA‐PEG nanoparticle‐based drug delivery system is synthesized, effectively targeting HECTD2 in vivo. The NPs achieved tumor‐targeting, controlled‐release, and biocompatibility, making them a promising therapeutic strategy for mitigating lenvatinib resistance. This study identifies HECTD2 as a nanotherapeutic target for overcoming lenvatinib resistance, providing a theoretical basis and translational application for HCC treatment.

## Graphical abstract

HECTD2 serves as an E3 ubiquitin ligase of KEAP1 to facilitate the antioxidative response and contributes to lenvatinib resistance in hepatocellular carcinoma. Lactylation of histone 3 on lysine residue 18 drives HECTD2 transcription. Targeting HECTD2 using PLGA‐PEG‐based nanoparticles offers novel strategies to overcome lenvatinib resistance in hepatocellular carcinoma.

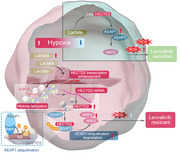

## 1 Introduction

Primary liver cancer ranks as the sixth most frequently diagnosed cancer and the third leading cause of cancer‐related death worldwide.^[^ ^1^ ^]^ Hepatocellular carcinoma (HCC) accounts for over 80% of primary liver cancer cases.^[^ ^2^ ^]^ Surgical resection and liver transplantation remain the only curative treatments for patients with HCC. However, most HCC cases are diagnosed at an advanced stage, rendering them ineligible for surgery. Lenvatinib, a multi‐targeted tyrosine kinase inhibitor that effectively targets VEGFR1 to VEGFR3, FGFR1 to FGFR4, PDGFRα, KIT, and RET, has been approved as a first‐line therapeutic agent for advanced HCC since 2018.^[^ ^3^ ^]^ However, drug resistance remains a major obstacle to its therapeutic efficacy. Many patients develop either acquired or intrinsic lenvatinib resistance, significantly limiting its clinical application and effectiveness.^[^ ^4^ ^]^ Therefore, understanding the molecular mechanisms underlying lenvatinib resistance is crucial for developing effective therapeutic strategies and improving patients with HCC prognosis.

HECT domain E3 ubiquitin protein ligase 2 (HECTD2), a member of the HECT‐type E3 ligase family members, was first identified as a susceptibility gene for neurodegenerative disorders.^[^ ^5^, ^6^ ^]^ As a relatively understudied member of the HECT E3 ligase family, HECTD2 has recently been implicated in diverse roles in human cancers. Downregulation of HECTD2 has been shown to facilitate prostate cancer growth.^[^ ^7^ ^]^ Whereas its upregulation promotes proteasomal degradation of euchromatic histone lysine methyltransferase 2, thereby suppressing colorectal cancer progression.^[^ ^8^ ^]^ Additionally, HECTD2 accelerates the cell cycle and plays a dominant role in melanoma progression.^[^ ^9^ ^]^ Furthermore, overexpression of HECTD2 promotes inflammatory responses, proliferation, and migration in renal cell carcinoma cells.^[^ ^10^ ^]^ Intriguingly, HECTD2 exhibits both tumor‐suppressive and oncogenic properties across different malignancies, adding to its biological complexity. Our previous bioinformatics analysis has comprehensively described the landscape of HECT E3 ligases in HCC and demonstrated an association between HECTD2 upregulation and poor survival outcomes.^[^ ^11^ ^]^ However, its specific substrates and mechanisms of action in HCC remain largely unknown.

Posttranslational modifications of histones constitute a key component of the hierarchy of epigenetic regulatory mechanisms. Lactate‐derived histone lactylation has been identified as a direct promoter of gene transcription across multiple biological processes.^[^ ^12^ ^]^ As hallmarks of cancer, the hypoxic microenvironment and enhanced glycolysis contribute to lactate accumulation.^[^ ^13^ ^]^ Histone lactylation has been demonstrated as a common type of posttranslational modification in HCC.^[^ ^14^ ^]^ Recent studies have also suggested that histone lactylation can modulate drug resistance in colorectal cancer,^[^ ^15^ ^]^ bladder cancer,^[^ ^16^ ^]^ and glioblastoma.^[^ ^17^ ^]^ However, the involvement of histone lactylation in the HCC response to lenvatinib remains unclear.

Here, we established lenvatinib‐resistant HCC cell lines, patient‐derived organoids, and xenografts models, and identified that the significant upregulation of HECTD2 was closely associated with resistance to lenvatinib. Gain‐of‐function and loss‐of‐function experiments verified that HECTD2 contributes to lenvatinib resistance both in vitro and in vivo. Mechanistically, HECTD2 functions as an E3 ubiquitin ligase to facilitate the ubiquitination of The Kelch‐like ECH‐associated protein 1 (KEAP1), leading to the activation of the antioxidative response and limiting the sensitivity of HCC cells to lenvatinib. Lactylation of histone 3 on lysine residue 18 (H3K18la) induces the upregulation of HECTD2 in lenvatinib‐resistant HCC cells. More importantly, using patient‐derived xenograft models and DEN/CCl_4_ induced HCC models, we demonstrated that nanoparticles (NPs) targeting HECTD2 may provide a novel therapeutic approach to mitigating lenvatinib resistance in HCC.

## 2 Results

### 2.1 HECTD2 Expression Level is Associated with Lenvatinib Resistance in HCC

**Figure 1:**
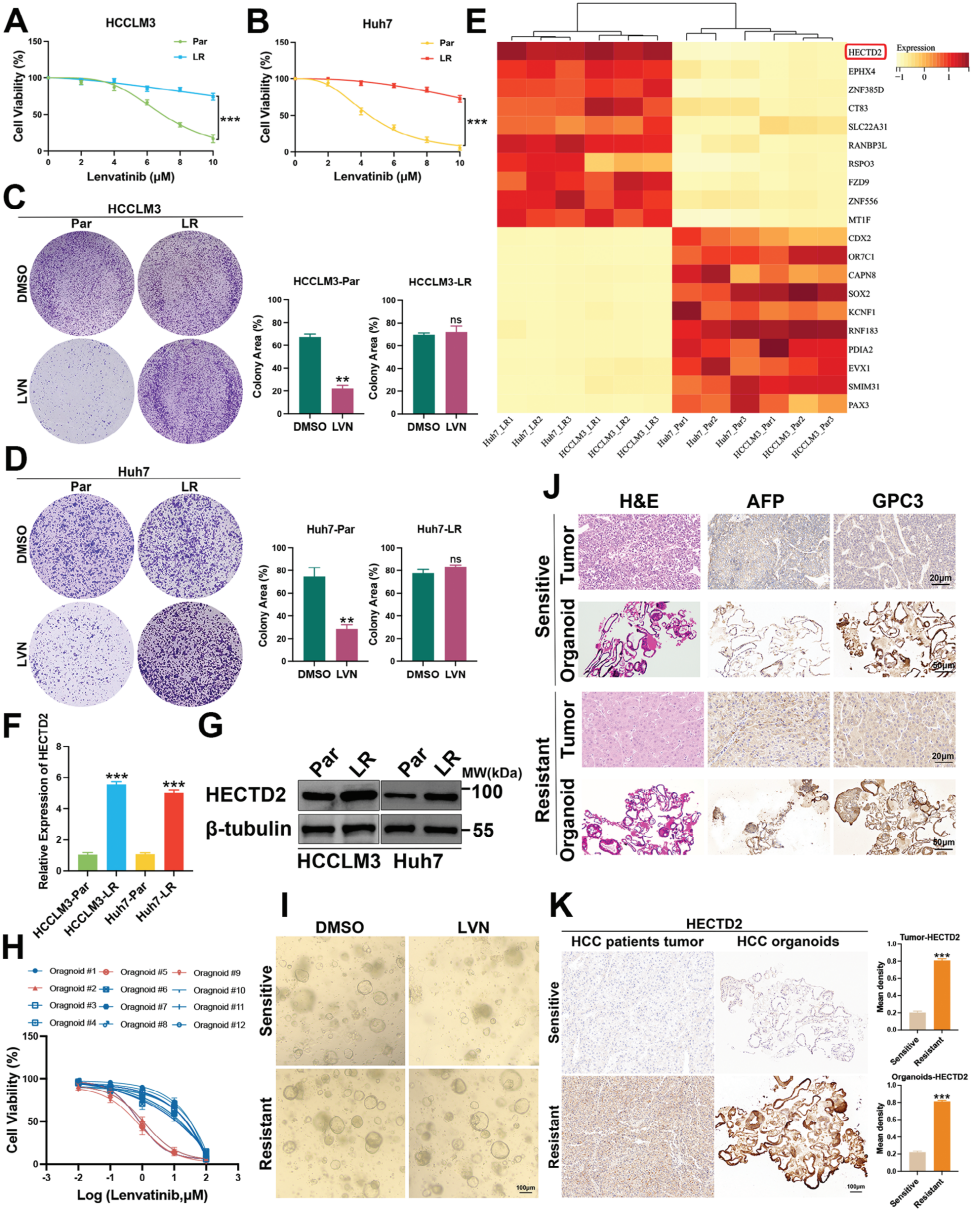
HECTD2 expression level is associated with lenvatinib resistance in HCC. A,B) Lenvatinib‐resistant HCCLM3 and Huh7 cells (HCCLM3‐LR and Huh7‐LR) and the corresponding parental cells (HCCLM3‐Par and Huh7‐Par) were treated with different doses of lenvatinib for 3 days and cell viability was examined to compare the half‐maximal inhibitory concentration (IC_50_) values between lenvatinib‐resistant cells and parental cells. The IC_50_ value of HCCLM3‐Par is 6.93 µm, IC_50_ of HCCLM3‐LR is 20.15 µm. The IC_50_ value of Huh7‐Par is 4.51 µm, IC_50_ of Huh7‐LR is 15.30 µm. C,D) HCCLM3‐LR, Huh7‐LR, and the corresponding parental cells were treated with lenvatinib (LVN) or DMSO for two weeks and colony formation assays were performed to compare the cell growth between lenvatinib‐resistant cells and parental cells. E) Unbiased proteomic screening of lenvatinib‐resistant and parental HCC cells showed that HECTD2 was among the top ten upregulated proteins in lenvatinib‐resistant HCC cells. F) qRT‐PCR was performed to show the mRNA expression levels of HECTD2 in lenvatinib‐resistant and parental HCC cells. G) Western blotting was conducted to assess the protein expression levels of HECTD2 in lenvatinib‐resistant and parental HCC cells. H) Therapeutic effects of lenvatinib on HCC organoids. Red lines indicated lenvatinib‐sensitive organoids (*n* = 3); Blue lines indicated lenvatinib‐resistant organoids (*n* = 9). I) Representative images of lenvatinib‐sensitive and lenvatinib‐resistant organoids. J) H&E evaluation and IHC staining with AFP and GPC3 of lenvatinib‐sensitive and lenvatinib‐resistant HCC patients and the corresponding organoids. K) IHC staining of HECTD2 in lenvatinib‐sensitive and lenvatinib‐resistant HCC patients and the corresponding organoids. **p < 0.01; ***p < 0.001; ns, no statistical significance.

Lenvatinib‐resistant HCC cell lines were established by exposing HCC cells to gradually increasing doses of lenvatinib and were subsequently referred to as HCCLM3 lenvatinib resistant (HCCLM3‐LR) and Huh7 lenvatinib resistant (Huh7‐LR). HCCLM3‐LR and Huh7‐LR exhibited higher IC_50_ values for lenvatinib compared to their parental cell lines (**Figure** **1**A,B). Colony formation assays further confirmed that HCCLM3‐LR and Huh7‐LR displayed reduced sensitivity to lenvatinib treatment (Figure 1C,D). To identify potential regulators of lenvatinib resistance in HCC, we performed unbiased proteomic screening of lenvatinib‐resistant and parental HCC cells (Figure S1A,B, Supporting Information). Cross‐referencing these results with the cancer genome atlas–liver hepatocellular carcinoma database (Figure S1C,D, Supporting Information), revealed that HECTD2 was among the top ten upregulated proteins in lenvatinib‐resistant HCC cells (Figure 1E). Quantitative real‐time polymerase chain reaction (qRT‐PCR) and western blotting further validated the significant upregulation of HECTD2 in HCCLM3‐LR and Huh7‐LR cells (Figure 1F,G).

To assess the relationship between HECTD2 expression and the efficacy of lenvatinib treatment in patients with HCC, we constructed organoids derived from patients with HCC and examined their response to lenvatinib treatment. As shown in Figure 1H, the majority of the organoids (75%, 9/12) exhibited resistance to lenvatinib, while 25% (3/12) remained sensitive to treatment. Furthermore, one lenvatinib‐resistant and one lenvatinib‐sensitive organoid were randomly selected for further analysis. The lenvatinib‐resistant organoid continued to grow robustly after 14 days of lenvatinib treatment (Figure 1I). Hematoxylin and Eosin (H&E) staining and immunohistochemistry (IHC) staining for AFP and GPC3 biomarkers in the selected organoids and the original patient tumor samples are shown in Figure 1J, confirmed that the organoids retained the same expression patterns as the original tumor tissues. HECTD2 protein expression level was markedly upregulated in lenvatinib‐resistant patient with HCC tissues and the corresponding organoids (Figure 1K). Bioinformatics further revealed that high HECTD2 expression was associated with poor patient overall survival (OS) and disease‐free survival (DFS) (Figure S1E,F, Supporting Information). qRT‐PCR results confirmed that HECTD2 mRNA expression was significantly elevated in lenvatinib‐resistant organoids and the corresponding patient tumor samples (Figure S2A,B, Supporting Information). Collectively, these findings demonstrate that HECTD2 is highly expressed in lenvatinib‐resistant HCC cell lines, organoids, and patient tissues, prompting further its functional role in HCC lenvatinib resistance.

### 2.2 HECTD2 Restricts the Response of HCC to Lenvatinib

**Figure 2:**
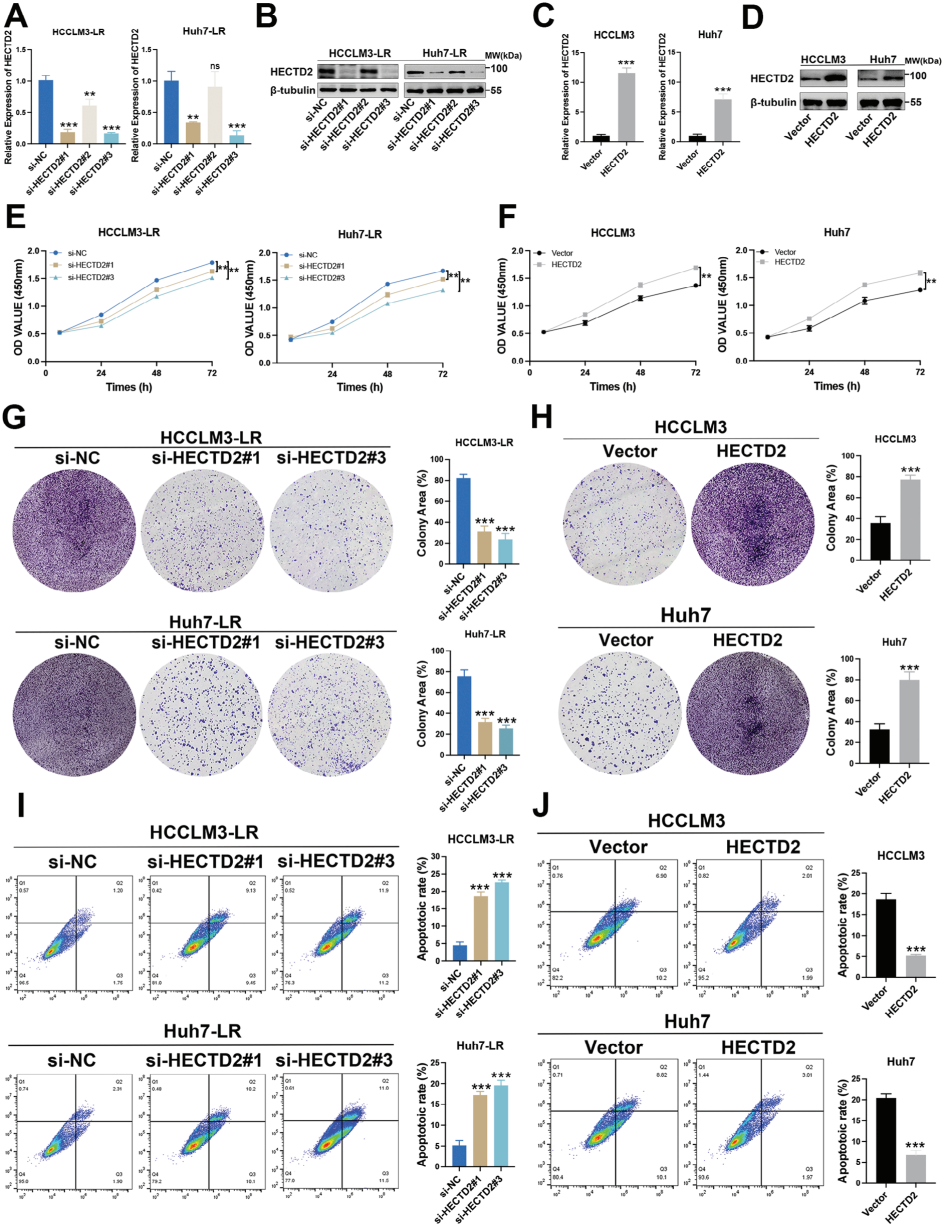
HECTD2 restricts the response of HCC to lenvatinib. A,B) Knockdown efficiency of HECTD2 in HCCLM3‐LR and Huh7‐LR cells was assessed using qRT‐PCR and western blotting. C,D) Overexpression efficiency of HECTD2 in HCCLM3 and Huh7 cells was assessed using qRT‐PCR and western blotting. E) The proliferative ability of HECTD2‐silenced HCCLM3‐LR and Huh7‐LR cells was evaluated using CCK‐8 assays. F) The proliferative ability of HECTD2‐overexpressing HCCLM3 and Huh7 cells was evaluated using CCK‐8 assays. G) Colony formation assays were performed to examine the cell proliferation of HECTD2‐silenced HCCLM3‐LR and Huh7‐LR cells. H) Colony formation assays were performed to examine the cell proliferation of HECTD2‐overexpressing HCCLM3 and Huh7 cells. I) Apoptosis rate of HCCLM3‐LR and Huh7‐LR cells with HECTD2 knockdown was evaluated by flow cytometry. J) Apoptosis rate of HCCLM3 and Huh7 cells with HECTD2 overexpression was evaluated by flow cytometry. All experiments were performed under lenvatinib treatment. **p < 0.01; ***p < 0.001; ns, no statistical significance.

To further elucidate the role of HECTD2 in modulating lenvatinib resistance, we knocked down HECTD2 in HCCLM3‐LR and Huh7‐LR cells and assessed the knockdown efficiency using qRT‐PCR and western blotting (**Figure** **2**A,B). si‐HECTD2#1 and si‐HECTD2#3 demonstrated the most significant knockdown efficiency and were selected for subsequent experiments. Additionally, HECTD2 was overexpressed in the lenvatinib‐sensitive HCCLM3 and Huh7 cell lines, with overexpression efficiency confirmed by qRT‐PCR and western blotting (Figure 2C,D). Cell Counting Kit‐8 (CCK‐8) assays were performed to assess the proliferative capacity of lenvatinib‐resistant HCC cells with HECTD2 knockdown and lenvatinib‐sensitive cells with HECTD2 overexpression. HECTD2 knockdown in HCCLM3‐LR and Huh7‐LR cells significantly reduced cell proliferation upon lenvatinib treatment, while HECTD2 overexpression increased proliferation in lenvatinib‐sensitive HCCLM3 and Huh7 cells (Figure 2E,F). Colony formation assays further confirmed that HECTD2 knockdown suppressed proliferation in HCCLM3‐LR and Huh7‐LR cells, whereas HECTD2 overexpression promoted cell growth in HCCLM3 and Huh7 cells (Figure 2G,H). Flow cytometry analysis revealed that HECTD2 knockdown significantly increased apoptosis rates in HCCLM3‐LR and Huh7‐LR cells (Figure 2I). In contrast, HECTD2 overexpression reduced apoptosis in lenvatinib‐sensitive HCCLM3 and Huh7 cells (Figure 2J). Collectively, these findings suggest that HECTD2 knockdown restores sensitivity to lenvatinib in resistant HCC cells, while HECTD2 overexpression reduces the vulnerability of lenvatinib‐sensitive HCC cells to lenvatinib treatment.

### 2.3 HECTD2 Interacts with KEAP1

**Figure 3:**
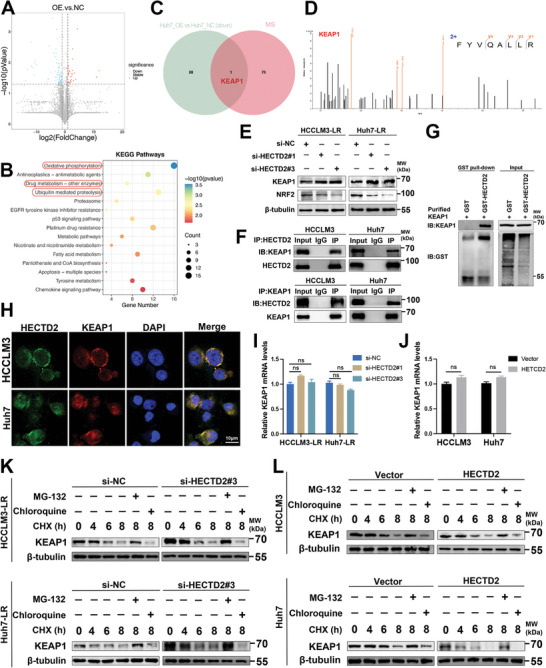
HECTD2 interacts with KEAP1. A) The differentially expressed protein profile in lenvatinib‐sensitive Huh7 cells with or without HECTD2 overexpression. B) HECTD2‐associated molecular events were demonstrated using KEGG analysis on the differentially expressed protein profile. C) Venn graph showing the differentially expressed protein profile upon HECTD2 overexpression and HECTD2‐interacting protein profile. D) Mass spectrometry result of KEAP1 from the immunoprecipitation products of HECTD2. E) Western blotting analysis was performed to detect the protein expression levels of KEAP1 and NRF2 in HECTD2‐silenced HCCLM3‐LR and Huh7‐LR cells. F) Co‐IP results showing the interaction between KEAP1 and NRF2 in HCC cells. G) GST‐pulldown assays showing that HECTD2 interacted with KEAP1. H) Immunofluorescence showing the colocalization of HECTD2 and KEAP1 in the cytoplasm. I,J) The mRNA expression levels of KEAP1 upon HECTD2 knockdown or overexpression. K,L) KEAP1 protein levels were evaluated in lenvatinib‐resistant and lenvatinib‐sensitive HCC cells after CHX, MG‐132, or chloroquine treatment. ns, no statistical significance.

To investigate the mechanism underlying HECTD2‐mediated lenvatinib resistance in HCC, we first performed protein sequencing of lenvatinib‐sensitive Huh7 cells with or without HECTD2 overexpression, establishing the landscape of differentially expressed proteins upon HECTD2 overexpression (**Figure** **3**A). Kyoto Encyclopedia of Genes and Genomes (KEGG) analysis of the differentially expressed proteins revealed that HECTD2‐associated molecular events were primarily enriched in oxidative stress, drug metabolism, and ubiquitination pathways (Figure 3B). To identify potential HECTD2‐binding proteins, mass spectrometry analysis was conducted on immunoprecipitated HECTD2 complexes. Cross‐referencing this dataset with the protein sequencing results revealed KEAP1 as the only candidate substrate of HECTD2 (Figure 3C,D). The KEAP1/ nuclear factor erythroid 2–related factor 2 (NRF2) system is a well‐conserved cellular defense mechanism against oxidative stress.^[^ ^18^ ^]^ Under quiescent conditions, KEAP1 sequesters NRF2 in the cytoplasm and targets it for proteasomal degradation. Upon exposure to cellular stress, NRF2 is related from KEAP1 repression, translocases to the nucleus, and activates the transcription of its target genes.^[^ ^19^ ^]^

To examine the relationship between HECTD2 and the KEAP1/NRF2 pathway, we measured the protein expression levels of KEAP1 and NRF2 in lenvatinib‐resistant HCC cell lines following HECTD2 knockdown. As shown in Figure 3E, KEAP1 expression was upregulated whereas NRF2 expression was downregulated upon HECTD2 silencing. To further verify the interaction between HECTD2 and KEAP1, co‐immunoprecipitation (co‐IP) and Glutathione‐S‐Transferase (GST)‐pulldown assays were performed (Figure 3F,G). Consistently, both assays confirmed a direct interaction between HECTD2 and KEAP1. Immunofluorescence analysis revealed colocalization of HECTD2 and KEAP1 in the cytoplasm (Figure 3H). Next, we investigated whether HECTD2 influences KEAP1 expression at the transcriptional or post‐translational level. Knockdown or overexpression of HECTD2 had no significant effect on KEAP1 mRNA levels (Figure 3I,J), indicating that HECTD2 does not regulate KEAP1 at the transcriptional level. Therefore, we hypothesized that HECTD2 may affect KEAP1 expression through protein degradation mechanisms. KEAP1 protein half‐life was assessed in HECTD2‐overexpressing and HECTD2‐silenced HCC cells following cycloheximide (CHX) treatment. The results demonstrated that HECTD2 overexpression accelerated KEAP1 protein degradation. Further analysis showed that KEAP1 protein levels were unaffected by the autophagy inhibitor chloroquine, but were significantly increased upon treatment with the proteasome inhibitor MG‐132 (Figure 3K,L). Collectively, these findings suggest that HECTD2 regulates KEAP1 protein stability through the ubiquitin‐proteasome pathway.

### 2.4 HECTD2 Serves as an E3 Ubiquitin Ligase of KEAP1

**Figure 4:**
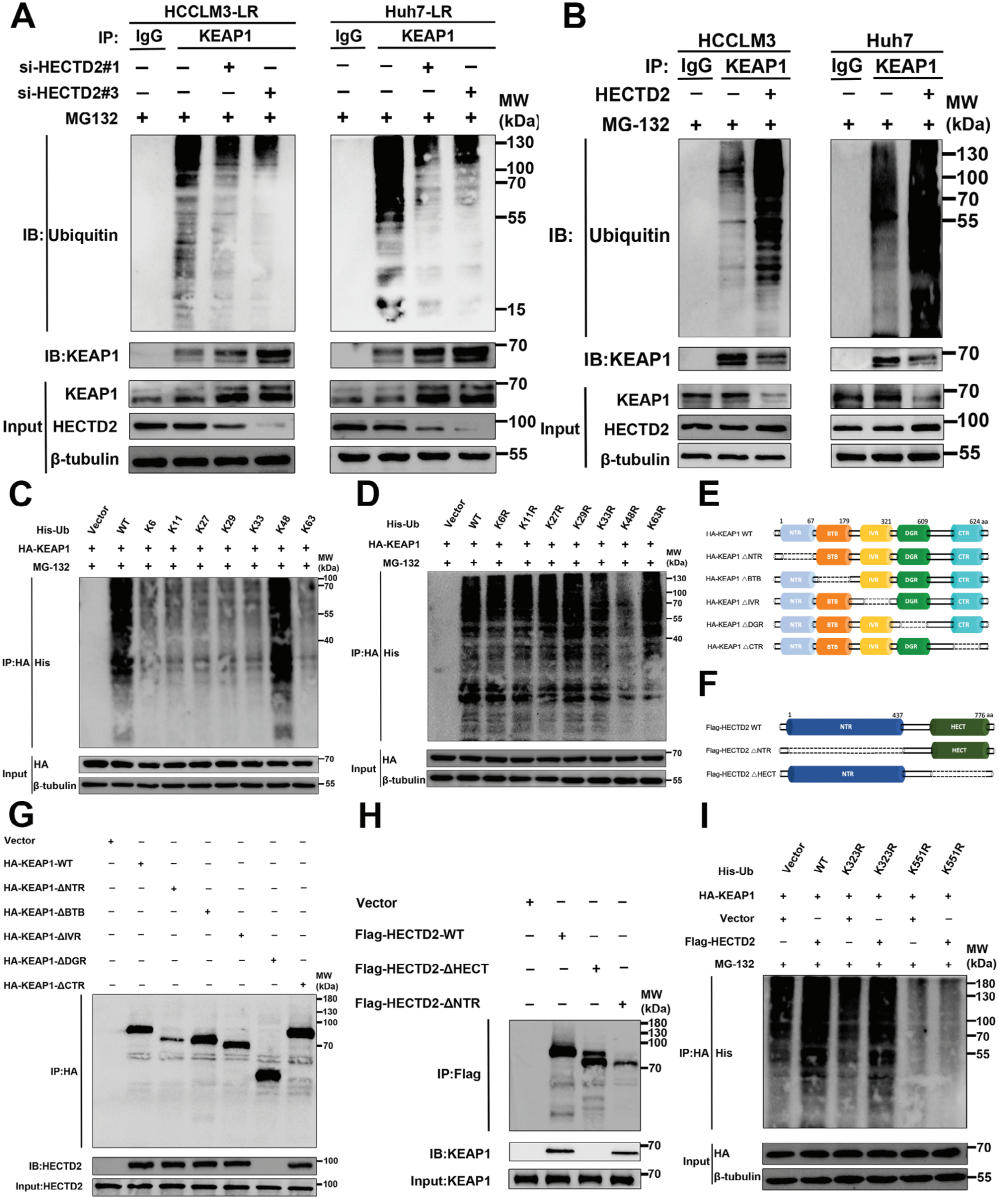
HECTD2 serves as an E3 ubiquitin ligase of KEAP1. A) KEAP1 ubiquitination and KEAP1 protein levels were examined in HCCLM3‐LR and Huh7‐LR cells with HECTD2 knockdown. B) KEAP1 ubiquitination and KEAP1 protein levels were assessed in HCCLM3 and Huh7 cells with HECTD2 overexpression. C,D) The specific type of polyubiquitination linkage of KEAP1 was determined by mutating each lysine residue or six out of the seven lysine residues of ubiquitin (K6, K11, K27, K29, K33, K48, and K63) to arginine. E) Different truncations of KEAP1. F) Different truncations of HECTD2. G) Interaction between HECTD2 and different truncations of KEAP1 was examined. H) Interaction between KEAP1 and different truncations of HECTD2 was evaluated. I) The specific KEAP1 lysine residue that mediated KEAP1 ubiquitination was investigated using K323 or K551 mutated KEAP1 protein.

Ubiquitination assays were conducted to assess the ubiquitination levels of KEAP1 in HCC cells with HECTD2 knockdown or overexpression. As shown in **Figure** **4**A,B, HECTD2 knockdown resulted in reduced KEAP1 ubiquitination and elevated KEAP1 protein levels, whereas HECTD2 overexpression enhanced KEAP1 ubiquitination and decreased KEAP1 protein levels. To determine the specific type of polyubiquitin linkage, each lysine residue or six of the seven lysine residues of ubiquitin (K6, K11, K27, K29, K33, K48, and K63) were mutated to arginine, and the ubiquitination levels of KEAP1 were compared (Figure 4C,D). The results indicated that HECTD2 promotes KEAP1 ubiquitination via K48‐linked polyubiquitin chains. To identify the domains of KEAP1 and HECTD2 responsible for their interaction, we constructed KEAP1 and HECTD2 truncation mutants (Figure 4E,F). As shown in Figure 4G,H, the DGR domain of KEAP1 and the HECT domain of HECTD2 were found to mediate their interaction. Rescue experiments using CCK‐8 and colony formation assays yielded consistent results, further supporting these findings (Figure S3A–D, Supporting Information). Moreover, sequence analysis revealed two lysine residues (K323 and K551) in the DGR domain of KEAP1. To identify the specific lysine residue mediating KEAP1 ubiquitination, KEAP1 mutants with K323R or K551R substitutions were generated. Ubiquitination assays revealed that HECTD2‐mediated KEAP1 ubiquitination occurred specifically at the K551 residue (Figure 4I). To investigate the biological relevance of these modifications, K323R and K551R mutant plasmids were transfected into lenvatinib‐resistant HCC cell lines for colony formation assays. The results demonstrated that cells transfected with the K551R mutant plasmid exhibited decreased sensitivity to lenvatinib (Figure S4A,B, Supporting Information), further confirming that KEAP1 ubiquitination by HECTD2 occurs at the K551 residue. Overall, these results demonstrate that HECTD2 functions as an E3 ubiquitin ligase for KEAP1, adding a K48‐linked polyubiquitin chain at the K551 residue of KEAP1.

### 2.5 HECTD2 Sustains Lenvatinib Resistance by Attenuating Oxidative Stress

**Figure 5:**
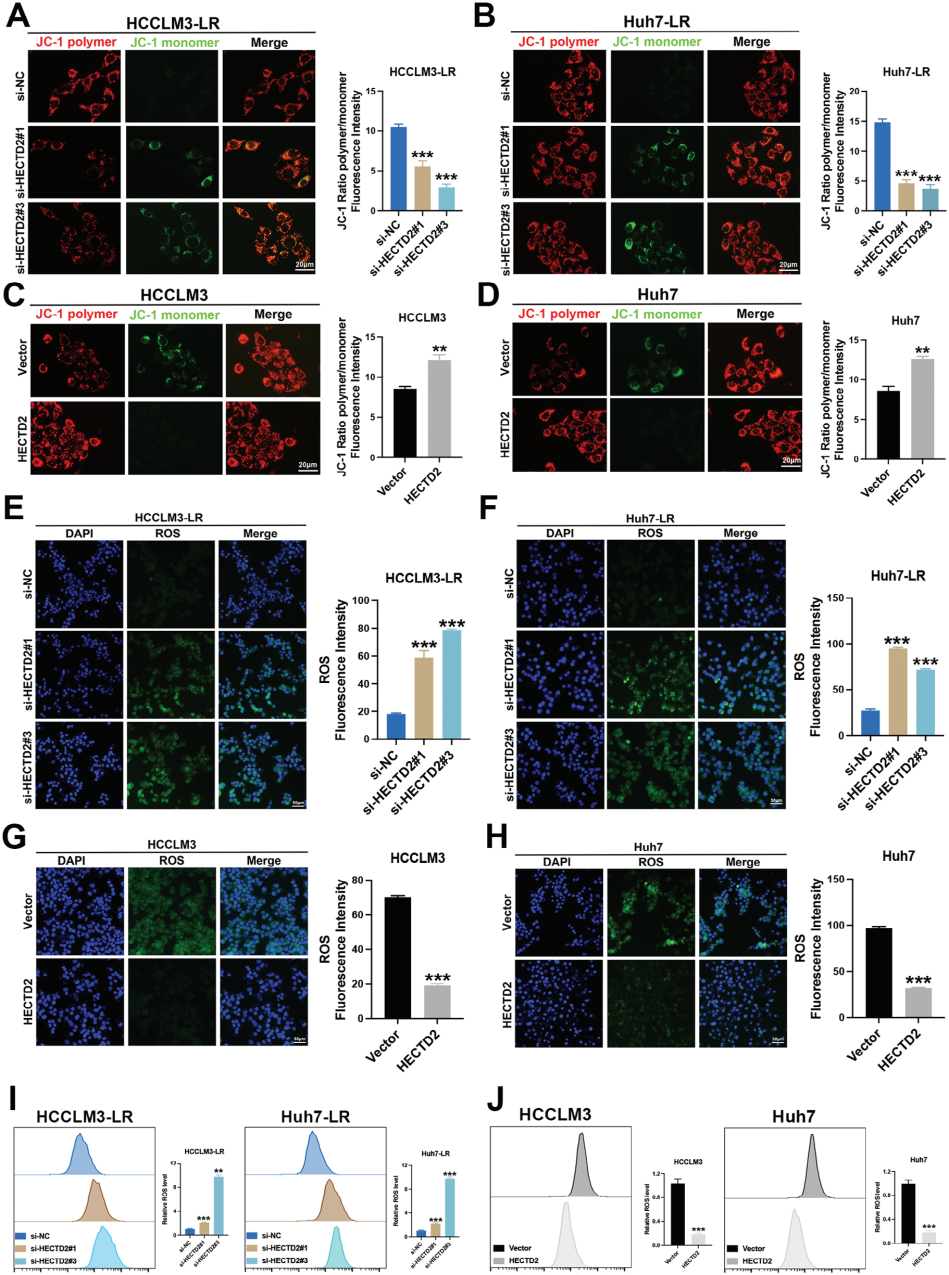
HECTD2 sustains lenvatinib resistance through attenuating oxidative stress. A,B) JC‐1 staining assays of HECTD2‐silenced HCCLM3‐LR and Huh7‐LR cells. C,D) JC‐1 staining assays of HECTD2‐overexpressed HCCLM3 and Huh7 cells. E,F) ROS levels of HECTD2‐silenced HCCLM3‐LR and Huh7‐LR cells. G,H) ROS levels of HECTD2‐overexpressed HCCLM3 and Huh7 cells. I) Flow cytometry assays were performed to compare the ROS levels of HECTD2‐silenced HCCLM3‐LR and Huh7‐LR cells. J) Flow cytometry assays were performed to compare the ROS levels of HECTD2‐overexpressed HCCLM3 and Huh7 cells. **p < 0.01; ***p < 0.001.

Since our previous findings indicated that HECTD2‐associated signaling pathways were primarily enriched in oxidative stress regulation (Figure 3B) and that the KEAP1/NRF2 signaling pathway was modulated by HECTD2 (Figure 3E), we further explored the role of oxidative stress in HECTD2‐mediated lenvatinib resistance. Mitochondrial membrane potential (MMP) was assessed using the ratio of red fluorescence from JC‐1 polymers to green fluorescence from JC‐1 monomers. The loss of MMP is commonly associated with drug‐induced tumor cell death. Lenvatinib treatment decreased the JC‐1 polymer/monomer ratio (Figure S5, Supporting Information), confirming its contribution to MMP loss. Furthermore, HECTD2 knockdown in lenvatinib‐resistant cells resulted in a shift from the JC‐1 polymer form to the JC‐1 monomer form, indicating decreased MMP (**Figure** **5**A,B). Conversely, HECTD2 overexpression increased MMP in HCC cells (Figure 5C,D). To further investigate oxidative stress, reactive oxygen species (ROS) levels were measured using a ROS fluorescent probe. HECTD2 knockdown significantly increased ROS levels (Figure 5E,F), while HECTD2 overexpression suppressed ROS generation (Figure 5G,H). These findings were corroborated using flow cytometry assays for ROS detection (Figure 5I,J). Immunofluorescence analysis was performed to assess the subcellular localization of NRF2 in both the cytoplasm and nucleus following lenvatinib treatment. As shown in Figure S6 (Supporting Information), NRF2 was predominantly localized in the nucleus of lenvatinib‐resistant HCC cell lines, whereas in lenvatinib‐sensitive cells, it was more abundant in the cytoplasm. In rescue experiments, NRF2 knockdown counteracted the increased cell growth (Figure S7A,B, Supporting Information) and decreased ROS levels (Figure S7C,D, Supporting Information) observed in HECTD2‐overexpressing cells. Collectively, these results demonstrate that HECTD2 limits oxidative stress by regulating the KEAP1/NRF2 system, thereby facilitating lenvatinib resistance in HCC.

### 2.6 HECTD2 Level is Clinically Relevant to HCC Prognosis and Lenvatinib Resistance

Using the median expression level of HECTD2 as a threshold, we categorized the enrolled 60 HCC patients received lenvatinib treatment into low and high HECTD2 expression groups. Kaplan–Meier survival analysis revealed that patients with high HECTD2 expression had significantly poorer overall survival (OS) and disease‐free survival (DFS) compared with those in the low‐expression group (Figure S8A,B, Supporting Information). Cox multivariate regression analysis further demonstrated that HECTD2 expression was an independent risk factor for both OS and DFS (Figure S8C,D, Supporting Information). IHC staining of HCC organoids and the corresponding primary tumor tissues showed that lenvatinib‐resistant organoids and tumor tissues exhibited elevated expression levels of HECTD2 and NRF2, along with reduced KEAP1 expression (Figure S8E, Supporting Information). IHC analysis of previously collected patient tissues confirmed a significant negative correlation between HECTD2 and KEAP1 expression levels and a positive correlation between HECTD2 and NRF2 expression levels (Figure S9, Supporting Information). Additionally, immunofluorescence analysis demonstrated high HECTD2 and low KEAP1 expression in lenvatinib‐resistant tumor tissues (Figure S10A, Supporting Information). Furthermore, lenvatinib‐resistant tumor tissues displayed high Ki‐67 expression and reduced terminal deoxynucleotidyl transferase dUTP nick‐end labeling (TUNEL) staining, indicating increased proliferation and decreased apoptosis, respectively (Figure S10B, Supporting Information).

### 2.7 HECTD2 Modulates KEAP1/NRF2 Signaling and Promotes Lenvatinib Resistance In Vivo

**Figure 6:**
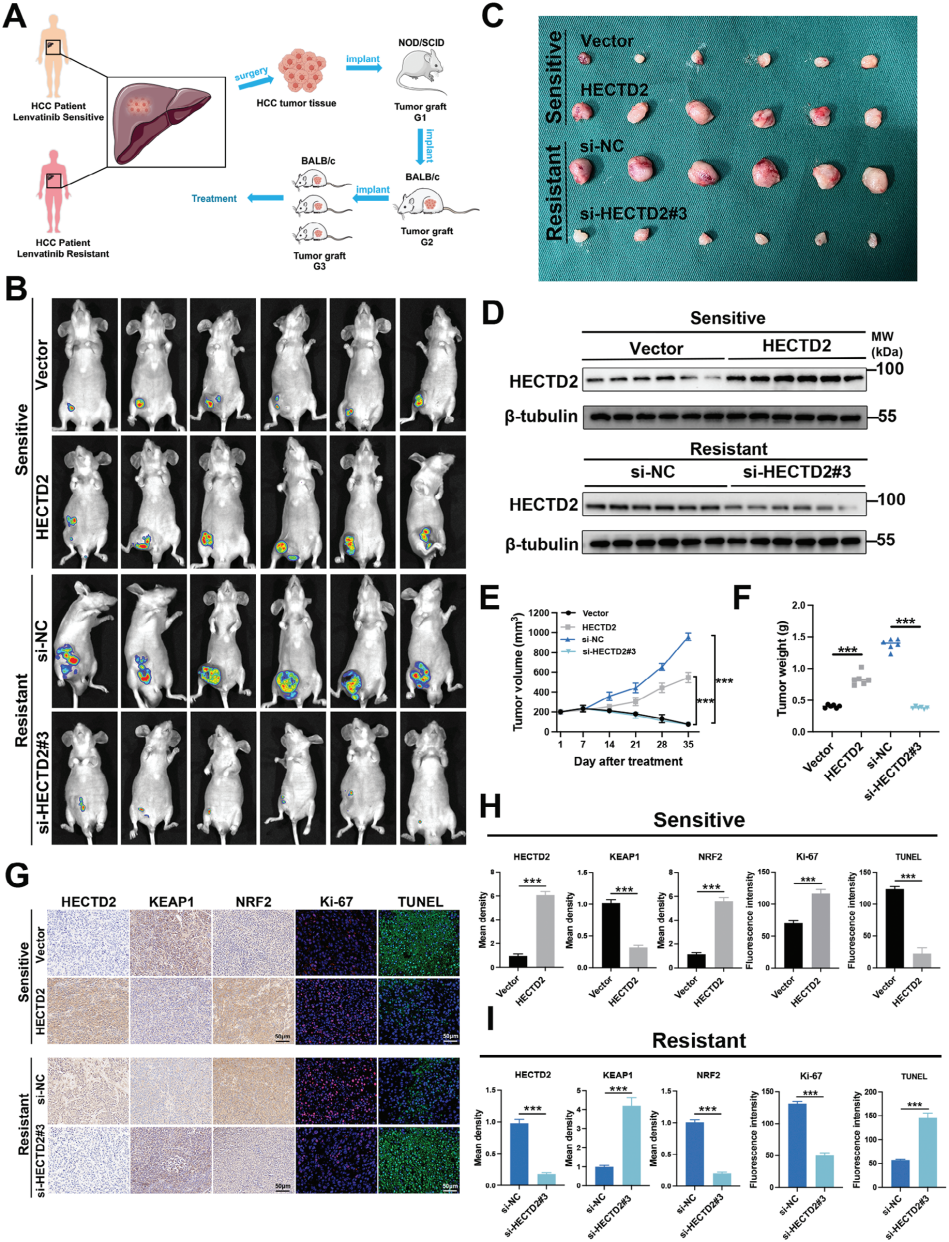
HECTD2 modulates KEAP1/NRF2 signaling and promotes lenvatinib resistance in vivo. A) Construction of lenvatinib‐sensitive and lenvatinib‐resistant PDX models. B) In vivo imaging of the xenografts from lenvatinib‐sensitive PDX models with HECTD2 overexpression and lenvatinib‐resistant PDX models with HECTD2 knockdown. C) Photographs of the tumors from lenvatinib‐sensitive PDX models with HECTD2 overexpression and lenvatinib‐resistant PDX models with HECTD2 knockdown. D) HECTD2 protein level of the PDX models. E) Tumor volume curves of the PDX models. F) Tumor weight of the PDX models. G) IHC staining results of HECTD2, KEAP1, NRF2 in the xenografts, and immunofluorescence results showing Ki‐67 and TUNEL of the xenografts. H,I) Quantitative results of HECTD2, KEAP1, NRF2, Ki‐67 and TUNEL in the xenografts. ***p < 0.001.

To further investigate the effects of HECTD2 on lenvatinib resistance, we conducted in vivo experiments using patient‐derived xenograft (PDX) models (**Figure** **6**A). Lenvatinib‐sensitive and lenvatinib‐resistant patient with HCC tissues were obtained and used to construct PDX models as previously described.^[^ ^20^ ^]^ We overexpressed HECTD2 in lenvatinib‐sensitive PDX models and silenced HECTD2 in lenvatinib‐resistant models. As shown in Figure 6B–F, HECTD2‐overexpressing lenvatinib‐sensitive PDX models developed significantly larger and heavier tumors than the vector control group. Conversely, tumors in HECTD2‐silenced lenvatinib‐resistant PDX models were significantly smaller and lighter than those in the si‐NC control group. IHC staining revealed higher expression levels of NRF2 and Ki‐67 and lower KEAP1 expression in the HECTD2‐overexpressing xenografts. In contrast, xenografts with HECTD2 knockdown displayed lower NRF2 and Ki‐67 expression and elevated KEAP1 levels (Figure 6G–I). TUNEL assays further demonstrated that HECTD2 overexpression suppressed cell apoptosis, while HECTD2 knockdown promoted apoptosis. Immunofluorescence analysis of HECTD2 and KEAP1 in xenografts derived from both lenvatinib‐sensitive and lenvatinib‐resistant PDX models showed that high HECTD2 expression correlated with low KEAP1 expression (Figure S11, Supporting Information). Collectively, these results demonstrate that high HECTD2 expression is associated with reduced KEAP1 and increased NRF2 expression levels, contributing to lenvatinib resistance in PDX models.

### 2.8 Targeting HECTD2 Using PLGA‐PEG(si‐HECTD2#3) NPs Effectively Retards Lenvatinib Resistance In Vivo

**Figure 7:**
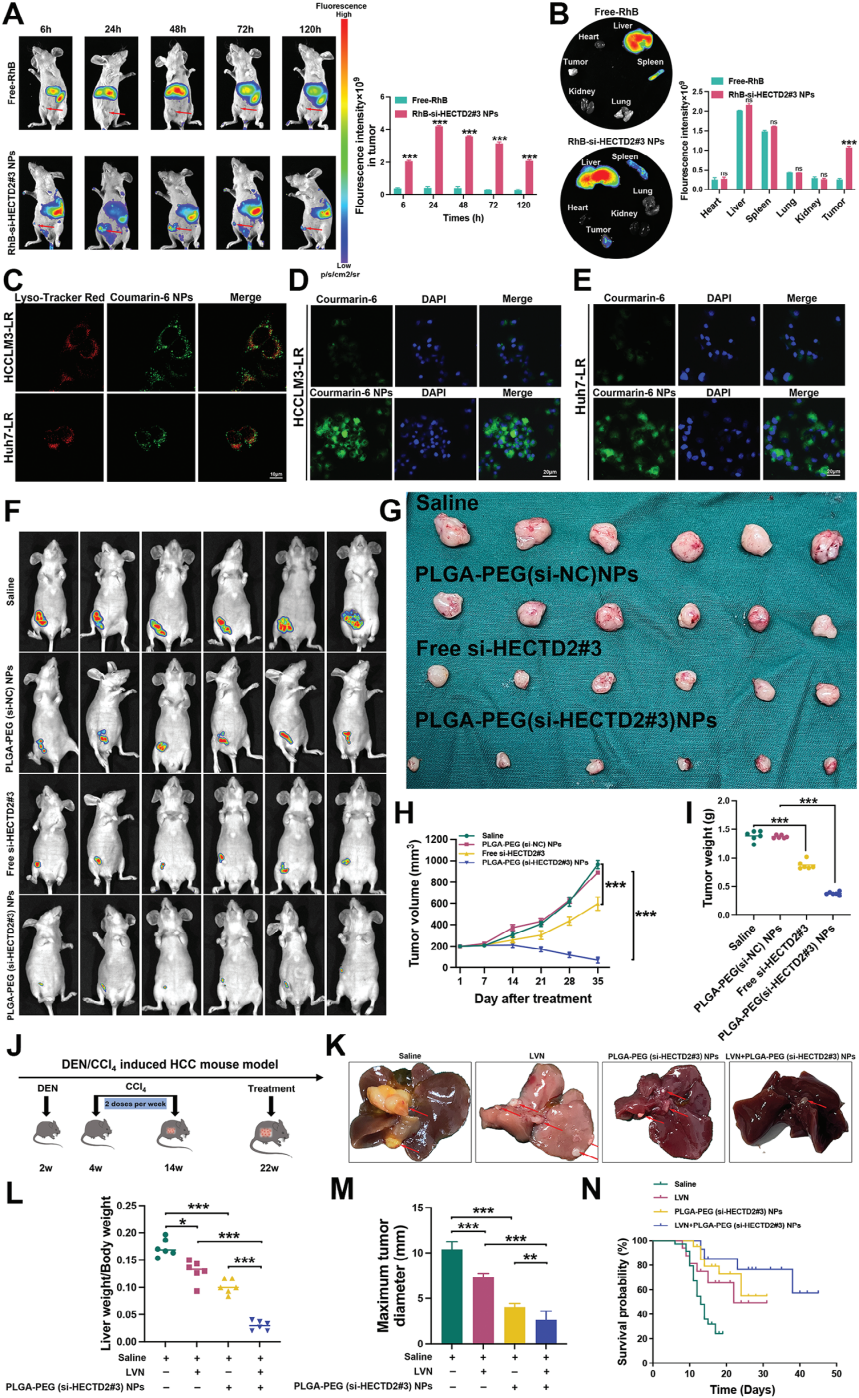
Targeting HECTD2 using PLGA‐PEG(si‐HECTD2#3) NPs effectively retards lenvatinib resistance in vivo. A) The accumulation of free‐RhB and RhB‐NPs at the tumor sites were visualized and quantified. B) The accumulation of free‐RhB and RhB‐NPs in the heart, liver, spleen, lung, kidney, and tumor. C) Lysosomal escape experiments were performed to evaluate the effects of NPs on the escape of loaded drug from lysosomes in HCCLM3 and Huh7 cells. D,E) The effects of NPs on cellular uptake of siRNAs in HCCLM3 and Huh7 cells. F) In vivo imaging of the xenografts treated with saline, PLGA‐PEG(si‐NC) NPs, free si‐HECTD2#3, and PLGA‐PEG(si‐HECTD2#3) NPs. G) Photographs of the tumors treated with saline, PLGA‐PEG(si‐NC) NPs, free si‐HECTD2#3, and PLGA‐PEG(si‐HECTD2#3) NPs. H) Volume curves of the tumors from different groups. I) Weight of the tumors from different groups. J) Construction of DEN/CCl_4_ induced HCC mouse model. K) Representative photographs of tumors from different groups. L) Ratios of liver to body weight (%). M) Maximum diameter of tumors from different groups. N) Survival analysis of the mice from different groups. *p < 0.05; **p < 0.01; ***p < 0.001.

We attempted to develop an effective drug delivery system for siRNAs targeting HECTD2. Naked siRNAs degrade easily and have difficulty penetrating cellular membranes, limiting their clinical application.^[^ ^21^ ^]^ We used a two‐emulsion solvent diffusion method to prepare PEGylated poly lactic‐co‐glycolic acid (PLGA) NPs loaded with si‐HECTD2#3 and arginine, referred to as PLGA‐PEG (si‐HECTD2#3) NPs. The PLGA‐PEG (si‐HECTD2#3) NPs obtained were milky white in solution (Figure S12A, Supporting Information). Transmission electron microscopy imaging confirmed the spherical morphology of the PLGA‐PEG (si‐HECTD2#3) NPs (Figure S12B, Supporting Information). The average particle size was measured at 139.9 nm (Figure S12C, Supporting Information) with a zeta potential of −7.92 mV (Figure S12D, Supporting Information). The encapsulation efficiency (EE%) of the PLGA‐PEG (si‐HECTD2#3) NPs was determined by spectrophotometry,^[^ ^22^ ^]^ calculated using the formula: EE% = (amount of encapsulated siRNA/amount of siRNA loaded) × 100%. The EE% was measured at 83.05% ± 1.50%. To examine the biodistribution of the NPs in vivo, free Rhodamine B (RhB) and RhB‐labeled si‐HECTD2#3 NPs were injected through the tail vein. As shown in **Figure** **7**A,B, RhB‐labeled si‐HECTD2#3 NPs enhanced RhB accumulation at tumor sites, confirming their targeted delivery capabilities. Further, to assess lysosomal escape efficiency, Courmarin‐6‐labeled NPs were utilized. The lack of colocalized fluorescence between NPs and lysosomes suggested that the NPs could escape from lysosomes (Figure 7C). The cellular uptake of siRNAs was investigated by labeling si‐HECTD2#3 NPs with Courmarin‐6 and incubating them with HCCLM3‐LR and Huh7‐LR cells. As shown in Figure 7D,E, PLGA‐PEG (si‐HECTD2#3) NPs significantly enhanced cellular uptake of the siRNA cargo compared to free siRNA. The release kinetics of si‐HECTD2#3 from the NPs were also examined (Figure S12E, Supporting Information). After 24 h, the cumulative release was ≈80% for free si‐HECTD2#3 and 40% for PLGA‐PEG (si‐HECTD2#3) NPs, with a sustained release pattern lasting up to 1 week. To assess nanoparticle stability, the particle size and PDI were monitored in fetal bovine serum (FBS), phosphate‐buffered saline (PBS), and water for over 8 days. The results indicated minimal variation, demonstrating the NPs' excellent stability in vitro (Figure S12F–H, Supporting Information).

Subsequently, in vivo evaluation of PLGA‐PEG (si‐HECTD2#3) NPs was conducted using a PDX model. Mice were treated with tail vein injections of saline, PLGA‐PEG (si‐NC) NPs, free si‐HECTD2#3, or PLGA‐PEG (si‐HECTD2#3) NPs. Tumor growth was significantly suppressed in the PLGA‐PEG (si‐HECTD2#3) NPs treatment group compared to the other groups (Figure 7F,G). The volume and weight of xenograft tumors in mice treated with PLGA‐PEG (si‐HECTD2#3) NPs were significantly reduced (Figure 7H,I). To assess the antitumor efficacy of the PLGA‐PEG (si‐HECTD2#3) NPs, colony formation assays, and flow cytometry were performed. The results indicated that in two lenvatinib‐sensitive HCC cell lines, the sensitivity to lenvatinib was significantly enhanced in the PLGA‐PEG (si‐HECTD2#3) NPs group compared to the DMSO control group. Furthermore, the combination of lenvatinib with PLGA‐PEG (si‐HECTD2#3) NPs exhibited the strongest therapeutic effect (Figure S13A, Supporting Information), suggesting a promising approach for clinical treatment strategies. Consistently, flow cytometry revealed that the percentage of apoptotic cells was highest in the lenvatinib combined with PLGA‐PEG (si‐HECTD2#3) NPs group (Figure S13B, Supporting Information), aligning with the colony formation assay results. To evaluate potential toxicity, H&E staining was performed on major organs (heart, liver, spleen, lung, and kidney) following intravenous administration of PLGA‐PEG (si‐HECTD2#3) NPs. No significant toxicity was observed (Figure S14A, Supporting Information). Additionally, serum levels of alanine aminotransferase (ALT), aspartate aminotransferase (AST), creatinine (CRE), and blood urea nitrogen (BUN) showed no significant differences among the treatment groups (Figure S14B, Supporting Information). Moreover, a chemically induced HCC mouse model was established for further validation (Figure 7J). The combination of PLGA‐PEG (si‐HECTD2#3) NPs with lenvatinib exhibited the most significant inhibitory effect on HCC tumor growth compared to the saline, lenvatinib alone, and PLGA‐PEG (si‐HECTD2#3) NPs groups (Figure 7K–M). Additionally, this combination therapy demonstrated the highest survival rate among the groups tested (Figure 7N). In conclusion, PLGA‐PEG (si‐HECTD2#3) NPs effectively target HECTD2 and overcome lenvatinib resistance in vivo, supporting their potential for clinical application in HCC treatment.

### 2.9 Histone Lactylation Drives HECTD2 Transcription

**Figure 8:**
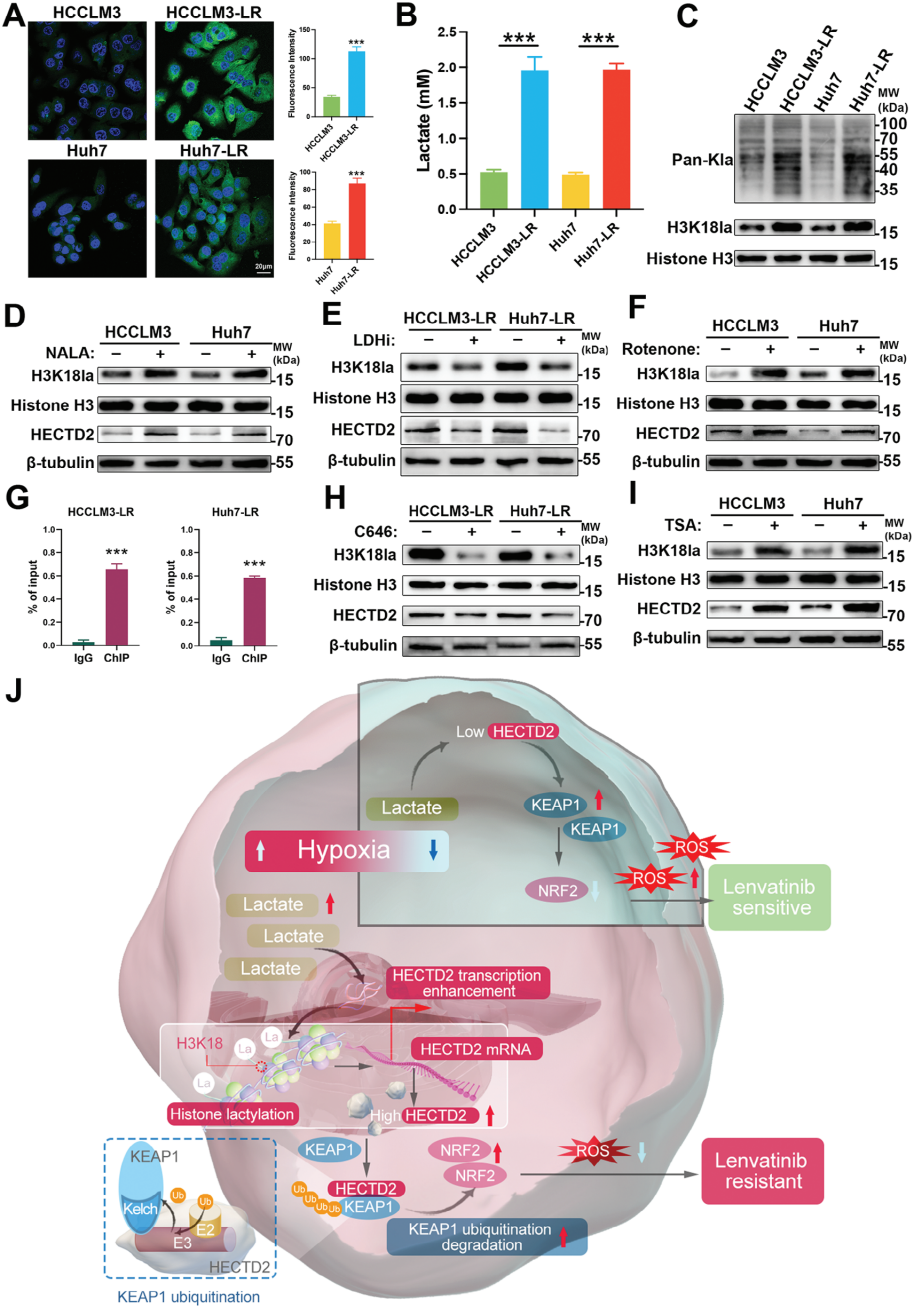
Histone lactylation drives HECTD2 transcription. A) Pimonidazole was used for hypoxia detection in lenvatinib‐sensitive HCCLM3 and Huh7 cells and HCCLM3‐LR and Huh7‐LR cells. B) Lactate levels in lenvatinib‐sensitive HCCLM3 and Huh7 cells and HCCLM3‐LR and Huh7‐LR cells. C) Pan‐Kla and H3K18la levels in lenvatinib‐sensitive HCCLM3 and Huh7 cells and HCCLM3‐LR and Huh7‐LR cells. D) After administration of NALA, H3K18la and HECTD2 levels in HCCLM3 and Huh7 cells were examined. E) H3K18la and HECTD2 levels in HCCLM3‐LR and Huh7‐LR cells were investigated after treatment of LDHi. F) Rotenone was added to HCCLM3 and Huh7 cells, and the levels of H3K18la and HECTD2 were detected. G) ChIP‐qPCR assays were performed to show the enrichment of H3K18la in the HECTD2 promoter region in HCCLM3‐LR and Huh7‐LR cells. H) After administration of C646, H3K18la and HECTD2 levels in HCCLM3‐LR and Huh7‐LR cells were compared. I) H3K18la and HECTD2 levels in HCCLM3 and Huh7 cells were evaluated after TSA treatment. J) The schematic graph of this study. ***p < 0.001.

To explore the role of lactate‐derived histone lactylation in lenvatinib resistance, we used pimonidazole for hypoxia detection and observed a significantly elevated hypoxia level in lenvatinib‐resistant HCC cell lines compared to their parental controls (**Figure** **8**A). Additionally, HCCLM3‐LR and Huh7‐LR cells exhibited significantly increased lactate levels relative to their lenvatinib‐sensitive parental counterparts (Figure 8B). Using a pan‐lactylation antibody, we identified elevated protein lactylation levels in lenvatinib‐resistant cells. Notably, the level of H3K18la, but not other histone lactylation marks, was significantly higher in HCCLM3‐LR and Huh7‐LR cells (Figure 8C; Figure S15A, Supporting Information). Bioinformatics analysis of H3K18la chromatin immunoprecipitation (ChIP‐seq data from GSE207814 further revealed significant enrichment of H3K18la at the promoter region of HECTD2 (Figure S15B–D, Supporting Information). To experimentally validate the link between H3K18la and HECTD2 expression, we treated HCCLM3 and Huh7 cells with sodium lactate, which resulted in increased levels of both H3K18la and HECTD2 (Figure 8D). Conversely, treatment with an LDH inhibitor reduced H3K18la and HECTD2 levels in HCCLM3‐LR and Huh7‐LR cells (Figure 8E). Moreover, treatment with rotenone, a mitochondrial respiratory chain complex I inhibitor, led to an increase in both H3K18la and HECTD2 expression (Figure 8F). To confirm the direct regulatory effect of H3K18la on HECTD2 transcription, we performed ChIP‐qPCR assays, which demonstrated significant H3K18la enrichment at the HECTD2 promoter region (Figure 8G). Previous reports have identified p300 and histone deacetylase (HDAC) as the writer and eraser enzymes for the H3K18la modification, respectively.^[^ ^23^, ^24^, ^25^ ^]^ Consistent with these findings, treatment with the p300 inhibitor C646 reduced H3K18la and HECTD2 expression in HCCLM3‐LR and Huh7‐LR cells (Figure 8H), while the HDAC inhibitor TSA increased H3K18la and HECTD2 expression in HCCLM3 and Huh7 cells (Figure 8I). Collectively, these results demonstrate that H3K18la modification promotes HECTD2 transcription, contributing to lenvatinib resistance in HCC. A schematic summary of this study is illustrated in Figure 8J.

## 3 Discussion

Drug resistance remains a significant challenge in HCC treatment, contributing to the disease's high fatality rate.^[^ ^26^ ^]^ Lenvatinib has demonstrated substantial survival benefits and is widely recognized as a first‐line therapy for patients with advanced HCC.^[^ ^27^ ^]^ However, many patients with HCC fail to achieve long‐term benefits from lenvatinib due to primary or acquired resistance. To identify novel therapeutic targets for overcoming lenvatinib resistance, we conducted unbiased proteomic screening of lenvatinib‐resistant and lenvatinib‐sensitive HCC cells. This analysis revealed HECTD2 as a critical regulator of lenvatinib resistance.

HECTD2, a member of the HECT E3 ligase family, has been recognized for its diverse roles in physiological and pathophysiological processes. Using both in vitro and in vivo models, we observed a significant upregulation of HECTD2 expression in lenvatinib‐resistant HCC cell lines, patient‐derived organoids, PDX mouse models, and patient cancer tissues. These findings align with our previous bioinformatics analyses,^[^ ^11^ ^]^ which showed that high HECTD2 expression is associated with poor survival outcomes in patients with HCC. Survival analyses of our cohort further revealed that elevated HECTD2 levels serve as an independent predictor for OS and DFS. To investigate the functional role of HECTD2 in lenvatinib resistance, we performed knockdown experiments in lenvatinib‐resistant HCCLM3‐LR and Huh7‐LR cells and overexpressed HECTD2 in their corresponding parental lenvatinib‐sensitive cells. HECTD2 knockdown sensitized HCCLM3‐LR and Huh7‐LR cells to lenvatinib treatment, while HECTD2 overexpression reduced the response of parental HCCLM3 and Huh7 cells to lenvatinib. In vivo validation using PDX mouse models derived from lenvatinib‐sensitive and lenvatinib‐resistant HCC patient samples confirmed these results. Overexpressing HECTD2 in PDX models enhanced resistance to lenvatinib treatment, leading to increased tumor volume and weight. Conversely, knocking down HECTD2 in lenvatinib‐resistant PDX models increased sensitivity to lenvatinib, significantly inhibiting the growth of engrafted tumors upon treatment.

Lenvatinib, a frontline therapeutic agent for advanced liver cancer, has significantly prolonged progression‐free survival (PFS) compared to sorafenib.^[^ ^28^ ^]^ However, the emergence of drug resistance remains a critical challenge for researchers to address. Lu et al. demonstrated that enhanced glycolysis in lenvatinib‐resistant Hep3B and Huh7 cells led to lactate accumulation and lysine acetylation of IGF2BP3, which subsequently elevated the mRNA levels of PCK2 and NRF2, thereby enhancing the antioxidant defense system. The acetylated IGF2BP3‐PCK2‐SAM‐m6A axis sustained higher expression of PCK2 and NRF2, reinforced the antioxidant defense, and promoted lenvatinib resistance in HCC. In vivo, treatment with liposomes carrying siRNAs targeting IGF2BP3 or the glycolysis inhibitor 2‐DG restored sensitivity to lenvatinib. These findings underscore the link between metabolic reprogramming and epigenetic regulation, suggesting that targeting metabolic pathways could offer novel strategies to overcome lenvatinib resistance in HCC.^[^ ^29^ ^]^ Zhang et al. further explored the genetic and drug‐sensitivity heterogeneity in liver cancer organoids through exome and transcriptome sequencing. By performing large‐scale drug sensitivity testing of liver cancer‐targeting drugs, combined with transcriptome data and machine learning models, they identified high expression of the c‐JUN protein as a key factor contributing to drug resistance in liver cancer. Notably, c‐JUN mRNA levels were significantly positively correlated with the IC_50_ of lenvatinib, highlighting its potential role as a predictive biomarker for treatment response. IHC staining revealed that c‐JUN protein levels were elevated in lenvatinib‐resistant regions. Furthermore, cancerous tissues exhibited higher c‐JUN expression compared to adjacent non‐cancerous tissues, suggesting that c‐JUN could serve as a potential therapeutic target for cancer treatment. Mechanistically, JNK and Wnt/β‐catenin signaling pathways were identified as upstream regulators of c‐JUN, mediating lenvatinib resistance.^[^ ^30^ ^]^ Xu et al. further identified phosphorylated non‐muscle myosin heavy chain 9 (p‐MYH9) as being upregulated in HCC samples, where its expression was associated with poor prognosis and lenvatinib resistance. Mechanistic studies demonstrated that p‐MYH9 (Ser1943) deubiquitinates and stabilizes hypoxia‐inducible factor 1α (HIF‐1α) by recruiting ubiquitin‐specific protease 22 (USP22), thereby contributing to lenvatinib resistance. This study confirmed that the p‐MYH9/USP22 complex stabilizes HIF‐1α under normoxic conditions, providing a promising therapeutic target for HCC cells that have metastasized into the bloodstream following drug resistance. Interestingly, CX‐4945, a CK2 inhibitor targeting p‐MYH9, and S02, a USP22 inhibitor, were shown to effectively reverse lenvatinib resistance in HCC cells, presenting a novel therapeutic strategy for lenvatinib‐resistant HCC patients.^[^ ^31^ ^]^

The KEAP1/NRF2 signaling pathway plays a critical role in cellular defense against oxidative and electrophilic stress.^[^ ^32^ ^]^ Upon exposure to extrinsic or intrinsic oxidative stimuli, cells upregulate NRF2 levels by promoting KEAP1 degradation. Subsequently, NRF2 translocates into the nucleus and activates multiple downstream antioxidant genes to eliminate ROS. The central role of the KEAP1/NRF2 pathway in the cellular stress response makes it a crucial regulatory node in various diseases. Previous studies have demonstrated the involvement of the KEAP1/NRF2 system in drug resistance across several cancer types, including chemoresistance in gastric cancer cells,^[^ ^33^ ^]^ radiation resistance in lung cancers,^[^ ^34^ ^]^ and sorafenib resistance in clear cell renal cell carcinoma.^[^ ^35^ ^]^ Genome‐wide CRISPR/Cas9 screening further revealed that KEAP1 disruption reduced the sensitivity of HCC cells to sorafenib, lenvatinib, and regorafenib treatments.^[^ ^36^ ^]^ Consistent with these findings, our study demonstrated that reducing HECTD2 expression enhanced oxidative stress, leading to increased sensitivity to lenvatinib treatment. ROS generated during oxidative stress plays a key role in TKIs resistance in a variety of tumors. Recent studies have demonstrated that upregulation of BCAT1 and reprogramming of branched‐chain amino acid metabolism in non‐small cell lung cancer cells attenuates ROS accumulation and mediates resistance to EGFR TKIs.^[^ ^37^ ^]^ UBQLN1 upregulation in hepatocellular carcinoma cells induces PGC1β degradation in a non‐ubiquitinating manner to reduce ROS production after sorafenib treatment, leading to sorafenib resistance.^[^ ^38^ ^]^ Our study further proved that HECTD2 was responsible for lenvatinib resistance by attenuating oxidative stress and reducing ROS production.

In this study, we conducted high‐throughput screening and mass spectrometry analyses, identifying HECTD2 as a binding partner of KEAP1 and a regulator of its protein expression. KEGG analysis revealed that HECTD2‐mediated signaling pathways were primarily enriched in oxidative stress, drug metabolism, and ubiquitination processes. Further validation using GST‐pulldown, co‐IP, and immunofluorescence assays confirmed the direct interaction between HECTD2 and KEAP1. HECTD2 overexpression or knockdown did not significantly affect KEAP1 mRNA levels but instead regulated KEAP1 protein expression. Ectopic HECTD2 expression shortened the half‐life of KEAP1 protein, an effect that was reversed by MG‐132 treatment, suggesting proteasomal degradation involvement. In vivo ubiquitination assays further confirmed that HECTD2 knockdown inhibited KEAP1 ubiquitination, leading to increased KEAP1 protein levels, whereas HECTD2 overexpression enhanced KEAP1 ubiquitination and reduced its protein expression. Using domain‐specific mutations, we demonstrated a direct interaction between the HECT domain of HECTD2 and the DGR domain of KEAP1. Furthermore, site‐specific mutations established that HECTD2 mediates K48‐linked polyubiquitination of KEAP1 at the K551 residue. Collectively, these results identify KEAP1 as a novel substrate of the E3 ubiquitin ligase HECTD2 in HCC and clarify the role of HECTD2 in regulating KEAP1 protein stability.

Lactylation modification has been implicated in various biological processes, with histone lactylation playing fundamental regulatory role in gene transcription.^[^ ^39^ ^]^ However, its involvement in lenvatinib resistance remains unclear. In this study, we demonstrated that lenvatinib‐resistant HCC cells exhibited aggravated hypoxia, excess lactate accumulation, and elevated pan‐lactylation levels compared to lenvatinib‐sensitive HCC cells. Further investigation revealed that H3K18la modification was the upstream mechanism driving HECTD2 transcription in lenvatinib‐resistant HCC cells. H3K18la has previously been associated with enhanced transcription of target genes. For instance, in idiopathic pulmonary fibrosis, H3K18la facilitates the transcription of YTHDF1, promoting the expression of m^6^A‐modified NREP.^[^ ^40^ ^]^ Similarly, H3K18la has been shown to activate the transcription of NSUN2 in colorectal cancer,^[^ ^41^ ^]^ while increased H3K18la in neurons stimulates Piezo2 transcription.^[^ ^42^ ^]^ We established that H3K18la contributes to HECTD2 transcription in HCC, ultimately restricting the response to lenvatinib treatment. Our findings elucidate the upstream regulation of HECTD2 and provide evidence of the intertwined association between lactylation and ubiquitination in HCC lenvatinib resistance.

In recent decades, significant progress has been made in the synthesis and characterization of engineered NPs, contributing to the approval of several nanodrugs and the advancement of numerous promising candidates in clinical trials.^[^ ^43^, ^44^ ^]^ An effective siRNA delivery system requires multiple functional attributes, including siRNA protection, serum stability in the bloodstream, the ability to avoid nonspecific cellular binding and uptake, reduced clearance by the reticuloendothelial system (RES), low cytotoxicity, specific targeting to cells of interest, and efficient cytoplasmic release.^[^ ^45^ ^]^ Unmodified siRNA is prone to degradation by nucleases in the bloodstream, leading to a shortened half‐life in vivo. PLGA NPs can effectively encapsulate and protect siRNA, preventing nuclease degradation and thereby prolonging its half‐life in *vivo*.^[^ ^46^ ^]^ As versatile carriers, PLGA NPs enhance, siRNA delivery by enabling cellular uptake due to their optimal size and surface properties,^[^ ^47^ ^]^ which facilitate penetration of cell membranes and intercellular spaces, delivering siRNA directly into target cells.^[^ ^48^ ^]^ Furthermore, fluorescent labeling of siRNA (e.g., Cy5) combined with PLGA encapsulation allows for visual tracking of siRNA within cells and tissues. This visualization technology supports real‐time monitoring of siRNA delivery, distribution, and transport processes, providing a powerful tool for gene therapy research.^[^ ^49^ ^]^ In summary, PLGA‐encapsulated siRNA offers multiple advantages, including excellent biocompatibility, enhanced delivery efficiency, strong stability, controlled drug release, targeted delivery, and real‐time monitoring capabilities. These properties make PLGA‐based siRNA delivery systems highly promising for applications in gene therapy, disease research, and drug delivery.

We have previously developed several nanomaterial‐based drug delivery systems with tumor‐targeting, controlled‐release, and biocompatible properties for cancer treatment, all demonstrating satisfactory antitumor activity.^[^ ^20^, ^50^, ^51^ ^]^ Building on these functional and mechanistic findings, we proposed that HECTD2 could serve as a potential therapeutic target for cancer treatment. In this study, we utilized the Food and Drug Administration‐approved nanocarrier PLGA^[^ ^52^ ^]^ to synthesize PLGA‐PEG NPs loaded with siRNAs targeting HECTD2. The NPs were fully characterized and their biodistribution was evaluated in vivo. Using RhB‐NPs, we confirmed enhanced tumor‐site accumulation. Courmarin‐6‐NPs further demonstrated that the nanoparticles facilitated the escape of the loaded drug from lysosomes, while also increasing cellular uptake of the delivered siRNA. The PLGA‐PEG (si‐HECTD2#3) NPs exhibited sustained release over ≈1 week and remained stable in FBS, PBS, and water. To investigate the therapeutic of PLGA‐PEG (si‐HECTD2#3) NPs in mitigating lenvatinib resistance in vivo, we employed PDX mouse models established from HCC patient with HCC tissues. Tumor volume and weight were significantly reduced in the PLGA‐PEG (si‐HECTD2#3) NPs‐treated group compared to the saline control, the PLGA‐PEG(si‐NC) NPs group, and the free si‐HECTD2#3 group. Similar antitumor effects were observed in the DEN/CCl_4_ induced HCC mouse model. Biosafety assessment was conducted through H&E staining of the heart, liver, spleen, lung, and kidney, along with serum analysis of ALT, AST, CRE, and BUN levels. No significant toxicity was observed across the treatment groups. Collectively, these findings indicate that PLGA‐PEG (si‐HECTD2#3) NPs represent a promising candidate for mitigating lenvatinib resistance in HCC, with favorable biocompatibility and therapeutic efficacy. Certainly, our current research may still have certain limitations. For instance, the current delivery method involves the separate administration of lenvatinib and PLGA‐PEG nanoparticles. In recent years, there has been a growing body of research exploring the co‐encapsulation and delivery of chemotherapeutic agents with nanoparticles. Moving forward, we aim to further investigate this approach to refine our drug delivery strategies, thereby paving the way for more innovative and effective therapeutic solutions for liver cancer.

## 4 Conclusion 

In summary, this study demonstrates that HECTD2 functions as an E3 ubiquitin ligase, adding K48‐linked polyubiquitin chains at the K551 residue of KEAP1, thereby alleviating oxidative stress induced by lenvatinib treatment. Histone lactylation at H3K18la was shown to enhance HECTD2 transcription, while elevated HECTD2 expression served as an independent predictive marker for patient with HCC prognosis. Targeting HECTD2 with PLGA‐PEG (si‐HECTD2#3) NPs effectively countered lenvatinib resistance in HCC therapy. This study offers new insights into lenvatinib resistance mechanisms in HCC and suggests promising diagnostic and therapeutic strategies for clinical HCC treatment.

## 5 Experimental Section

### Patients

A total of 60 HCC patients who received lenvatinib treatment at the First Affiliated Hospital of Wannan Medical College from 2021 to 2023 were enrolled in the study. Lenvatinib treatment response was evaluated according to the Response Evaluation Criteria in Solid Tumors 1.1 (RECIST1.1).^[^ ^53^ ^]^ Lenvatinib sensitivity was defined as disease control, including complete remission (CR), partial remission (PR), and stable disease (SD), while lenvatinib resistance was defined as progressive disease (PD) based on RECIST1.1 guidelines.^[^ ^3^ ^]^ OS was defined as the time interval between radical treatment and death from any cause. DFS was defined as the time between treatment and the absence of disease recurrence or new cancer‐related problems. This study was conducted in accordance with the Declaration of Helsinki and was approved by the Ethics Committee of the First Affiliated Hospital of Wannan Medical College (Approval Number: 2021‐12).

### CCK‐8 Assay

The indicated cells were seeded into 96‐well plates at a density of 2 × 10^3^ cells per well. At the specified time points, 10 µL of CCK‐8 solution (RiboBio, Guangzhou, China) was added to 100 µL of culture medium. Following a 2‐h incubation in the dark, absorbance was measured at 450 nm using an automated microplate reader (BioTek Instruments, Inc., USA). For lenvatinib sensitivity testing, organoids were seeded in 96‐well plates with Matrigel and treated with increasing doses of lenvatinib. After 5 days, organoid viability was assessed using the CellTiter‐Glo assay (Thermo Fisher, USA) following the manufacturer's protocol.

### Establishment of Lenvatinib‐Resistant Cell Lines and Unbiased Proteomic Analysis

Lenvatinib‐resistant cell lines were established as previously described.^[^ ^54^ ^]^ To determine the half maximal inhibitory concentration (IC_50_) for lenvatinib in HCCLM3 and Huh7 cells, cells were treated with varying concentrations of lenvatinib in 96‐well plates. After 3 days of incubation, cell viability was assessed using the CCK‐8 assay. Subsequently, HCCLM3 and Huh7 cells were continuously exposed to lenvatinib concentrations slightly below their IC_50_ values, with the concentration increased by 1 µm per week until reaching 10 µm. After several months, lenvatinib‐resistant HCC cell lines were successfully established and termed HCCLM3‐LR and Huh7‐LR. The resistant cell lines were maintained in culture with 10 µm lenvatinib. Unbiased proteomic analysis of lenvatinib‐resistant and parental HCC cells was performed by Applied Protein Technology (Shanghai, China).

### qRT‐PCR

cDNA synthesis was performed using the FastKing gDNA Dispelling RT SuperMix (TIANGEN, Beijing, China), and qRT‐PCR was conducted with the SuperReal PreMix Plus (TIANGEN, Beijing, China) following the manufacturer's instructions. Relative RNA expression levels were calculated using the 2^−ΔΔCt^ quantification method. The primers used for qRT‐PCR amplification were HECTD2: Forward: AGTTCACCTGCACATCTTGTTT and Reverse: GCCTTCATTTCGGATGATGATGATGC; KEAP1: Forward: CATGCATTTTGGGGAGGTGG and Reverse: GCTGATGAGGGTCACCAGTT. qRT‐PCR primers were designed and synthesized by GenePharma (Shanghai, China).

### Western Blotting

Total protein from cells and tissues was extracted using RIPA lysis buffer (Beyotime, Shanghai, China) containing 1 mm PMSF. The lysates were incubated on ice for 30 min and then centrifuged at 12 000rpm for 15 min at 4 °C to collect the supernatant. Protein concentrations were measured using the BCA Protein Assay Kit (Beyotime, Shanghai, China). Protein samples were boiled at 95 °C for 10 min, separated by SDS‐PAGE (Beyotime, Shanghai, China), and transferred onto PVDF membranes (Merck Millipore, Germany). The membranes were incubated with primary antibodies overnight at 4 °C, followed by incubation with secondary antibodies for 2 h at room temperature. After washing, protein bands were visualized using a chemiluminescence detection system (Bio‐Rad, USA).

### Organoid Culture

Organoids were isolated and cultured following previously described protocols.^[^ ^55^, ^56^ ^]^ Fresh tissues were placed into a sampling vial containing an organ preservation solution, supplemented with organoid buffer containing double antibiotics and gentamicin. Mucous membranes and excess fat were carefully removed. The tissues were washed three times with organoid buffer in a petri dish and then cut into ≈1 mm^3^ pieces. The tissue fragments were digested with tissue digestion solution at 37 °C for 10–15 min, and the digestion was terminated by adding organoid buffer. The resulting cell suspension was filtered through a 100 µm pore size cell sieve into a fresh 15 mL centrifuge tube. A small portion of the filtrate was examined under a microscope to confirm the presence of distinct crypt‐like tissue structures. The cells were centrifuged at 1200 rpm for 5 min at 4 °C, and the supernatant was carefully removed. The pellet was washed once with PBS. An appropriate amount of cell culture medium was added to the cell pellet, and the cells were gently resuspended into a single‐cell suspension. The suspension was centrifuged again at 1200 rpm for 5 min at 4 °C. The tissue organoid isolation medium and PBS were pre‐warmed to room temperature. The supernatant was discarded, and the pellet was re‐suspended in matrix gel. A 50 µL droplet of the organoid‐matrix gel mixture was placed at the bottom of a pre‐warmed 24‐well low‐adsorption culture plate, with one droplet per well. The plate was incubated at 37 °C in a CO_2_ incubator for 30 min to allow the gel to solidify. After solidification, the preheated tissue organoid isolation medium was added to each well for continued culture.

### Apoptosis Assay

Cell apoptosis was assessed using the Annexin V‐FITC/PI Apoptosis Detection Kit (BestBio, China). Briefly, cells were seeded into six‐well plates with fresh medium and treated with 10 µm lenvatinib for 3 days. After treatment cells were harvested, suspended in binding buffer, and incubated with Annexin V‐FITC and PI for 15 min at room temperature in the dark. Apoptotic cells were quantified using flow cytometry, and FlowJo software was used for apoptosis rate analysis.

### Immunoprecipitation and Co‐IP Assay

For KEAP1 immunoprecipitation, cells were lysed in an SDS‐containing buffer. Following centrifugation, the supernatant was incubated with a KEAP1 antibody at 4 °C overnight. After washing, Protein A/G agarose beads (Santa Cruz, USA) were added and incubated for 2 h at 4 °C. The beads were then washed, boiled, and subjected to western blotting analysis.

For co‐IP assays, cell extracts were centrifuged at 12 000 rpm for 10 min at 4 °C. The supernatant was collected and incubated overnight at 4 °C with the indicated antibody or an IgG control. Protein A/G agarose beads (Santa Cruz, USA) were added and incubated for an additional 2 h at 4 °C. The immunoprecipitated proteins were washed, harvested by centrifugation, and analyzed by western blotting.

### Mass Spectrometry

Mass spectrometry analysis was performed using enzymatic digestion with endoproteinases, such as trypsin to break down protein samples into peptides. The digested peptide samples were then analyzed using a high‐resolution liquid chromatography‐mass spectrometry system and performed by Applied Protein Technology (Shanghai, China). The obtained mass spectrometry data were processed and matched against a protein database using PD3.0/MaxQuant software for protein identification. For sample preparation, the protein samples underwent in‐gel digestion for proteolytic cleavage. Peptide desalting was performed using a C18 cartridge, followed by peptide lyophilization and reconstitution in 10 µL of 0.1% formic acid solution for quantification using OD280 measurements. Each peptide sample was separated using an HPLC liquid chromatography system. Buffer A consisted of 0.1% formic acid in water, while Buffer B was composed of 0.1% formic acid in 84% acetonitrile/water. The chromatographic column was equilibrated with 95% Buffer A prior to sample injection and separated through an analytical column. After chromatographic separation, peptide samples were subjected to mass spectrometry analysis using a high‐resolution mass spectrometer in positive ion detection mode.

### GST‐Pulldown Assay

A GST‐pulldown assay was performed using the GST‐pulldown Kit (Thermo Fisher Scientific, USA) following the manufacturer's instructions. A prokaryotic plasmid encoding GST‐tagged HECTD2 (GST‐HECTD2) was designed and synthesized by GenePharma (Shanghai, China). Briefly, the decoy protein containing GST‐HECTD2 was incubated overnight with the prey protein containing KEAP1. The protein complexes were purified using elution buffers, and the resulting antibody bead complexes were analyzed by western blotting.

### Protein Half‐Life Assays and Chloroquine Treatment

To determine the half‐life of KEAP1, CHX chase assay was performed. CHX, a protein biosynthesis inhibitor, was added to the cell culture at a concentration of 10 µg mL^−1^ (Sigma–Aldrich, USA). Cells were harvested at the indicated time points, and protein abundance was measured using western blotting analysis. For autophagy inhibition, cells were treated with 50 µm chloroquine (Sigma–Aldrich, USA) to block autophagic degradation pathways.

### Ubiquitination Assay

To assess KEAP1 ubiquitination levels, cells were treated with 10 µm MG‐132 (Aladdin, Shanghai, China) to inhibit proteasomal degradation. Total proteins were extracted from the lysed cells, and immunoprecipitation was performed using an anti‐KEAP1 antibody, as described in Section “Immunoprecipitation and co‐IP assay.” The immunoprecipitated proteins were subsequently analyzed by western blotting to detect ubiquitination levels.

### JC‐1 Staining Assay

JC‐1 staining was performed using the Enhanced Mitochondrial Membrane Potential Assay Kit (Beyotime, Shanghai, China) following the manufacturer's protocol. Briefly, JC‐1 staining working solution was added to the cell culture, and cells were incubated at 37 °C for 20 min. Mitochondrial membrane potential was assessed using a fluorescence microscope.

### Detection of Reactive ROS

Cells were seeded at a density of 1 × 10⁵ cells per well in a six‐well plate and treated with 10 µm lenvatinib. DCFH‐DA (Beyotime, Shanghai, China) was diluted 1:1000 in serum‐free culture medium to a final concentration of 10 µm L^−1^. Cells were resuspended in the diluted DCFH‐DA solution and incubated for 20 min at 37 °C. After incubation, cells were washed thoroughly to remove excess DCFH‐DA that did not enter the cells. ROS levels were measured using fluorescence microscopy and flow cytometry.

### Animal Models

To establish PDX mouse models, NOD/SCID and BALB/c mice were used as previously described.^[^ ^57^ ^]^ Briefly, tumor tissues obtained from patients with HCC were preserved in ice‐cold culture medium containing 1% penicillin/streptomycin. The tissues were cut into 2–3 mm^3^ fragments and subcutaneously implanted into the flanks of NOD/SCID mice. Once the engrafted tumors reached a volume of 1–2 cm^3^, they were harvested and re‐implanted into BALB/c nude mice. When the engrafted tumors grew to ≈50 mm^3^, an intratumoral injection of ten optical density HECTD2 plasmids, empty plasmids, cholesterol‐conjugated HECTD2 siRNA, or negative control RNAi was performed every 3 days for 3 weeks. Tumor volume and weight were monitored regularly, and tumors were subjected to qRT‐PCR and IHC assays for further analysis.

For the DEN/CCl_4_ induced HCC mouse model, HCC was induced via intraperitoneal injection of DEN (25 mg kg^−1^) at week 2 postpartum, followed by biweekly intraperitoneal injections of 0.5 mL kg^−1^ of CCl_4_ dissolved in corn oil from week 4 to 14. Mice were assigned to different treatment groups at week 22. All animal care and experimental procedures were approved by the Animal Welfare and Ethics Committee of Wannan Medical College (Approval Number: LLSC‐2021‐109).

### Preparation of PLGA‐PEG Nanomaterials

PLGA‐PEG(si‐HECTD2#3) NPs were prepared using a double emulsion solvent diffusion method as previously described.^[^ ^54^ ^]^ si‐HECTD2#3 was first dissolved in DEPC water and mixed with spermidine at an N/P ratio of 8:1. The mixture was incubated at room temperature for 15 min to form the si‐HECTD2#3/spermidine complex. A total of 10 mg of PLGA‐PEG‐COOH (Daigang Biomaterial Co. Ltd., Jinan, China) was dissolved in 500 µL of dichloromethane (Aladdin, China). This solution was added dropwise to the si‐HECTD2#3/spermidine complex and emulsified using a probe sonicator. Next, 4 mL of polyvinyl alcohol (2.5%; Aladdin) was added dropwise to the primary emulsion and further emulsified using a probe sonicator. The resulting emulsion was stirred at room temperature for 4 h. The NPs were collected by centrifugation and washed with DEPC water. A Nano Particle Analyzer (Zetasizer Nano ZSE, UK) was used to evaluate the particle size, zeta potential, and PDI of the NPs.

### Lysosomal Escape Experiments

Cells were treated with Courmarin‐6‐labeled NPs (2 µg mL^−1^) for 1 h. To visualize lysosomes, LysoTracker Red was added. Colocalization of NPs and lysosomes was observed using a confocal microscope to assess lysosomal escape efficiency.

### Cellular Uptake of NPs

Cells were treated with Courmarin‐6 and Courmarin‐6‐labeled NPs (2 µg mL^−1^) for 1 h. DAPI was used to stain cell nuclei, and a fluorescence microscope was employed to visualize and assess the cellular uptake of the NPs.

### Hypoxia Detection

The Hypoxyprobe™‐1 Plus Kit (Hypoxyprobe, Inc., USA) was used to assess hypoxic levels in lenvatinib‐sensitive and lenvatinib‐resistant HCC cell lines following the manufacturer's protocol. Pimonidazole and FITC‐Mab1 were used for immunofluorescence‐based visualization of cellular hypoxia levels.

### Statistical Analysis

Data are presented as means ± standard deviation and were analyzed using the Prism software package (GraphPad, USA). Statistical significance was determined using Student's *t*‐test or two‐way ANOVA, as appropriate. A *p*‐value < 0.05 was considered statistically significant.

## Conflict of Interest

The authors declare no conflict of interest.

## Author Contributions

R.D., X.Z., Z.C., and J.C. initiated the project and designed the study. R.D., Y.F., Y.H., P.G., B.Z., M.Z., and Z.W. performed the experiments. L.W., S.W., and X.W. collected clinical specimens. R.D. and Z.C. wrote the manuscript. X.Z., J.C., and X.W. reviewed the manuscript. All authors read and approved the final manuscript.

## Ethics Approval and Consent to Participate

Human sample acquisition was approved by the Ethics Committee of the First Affiliated Hospital of Wannan Medical College, in accordance with the Declaration of Helsinki. All animal care and experiments were approved by the Animal Welfare and Ethics Committee of Wannan Medical College.

## Supporting information



Supplementary Information

## Data Availability

All the data that support the findings of this study are available within the article and supplemental information or available from the authors upon request.
