# Supplementary material for: Lactylation‐Driven HECTD2 Limits the Response of Hepatocellular Carcinoma to Lenvatinib
Source: Adv Sci (Weinh). 2025 Feb 20;12(15):2412559. doi: 10.1002/advs.202412559 (PMC12005811; doi:10.1002/advs.202412559)
Supplement: Supplementary file 1 — Supplementary Information [file ADVS-12-2412559-s001.docx]

Additional file 1: Supplementary methods

**Cell culture**

Huh7 and HCCLM3 cells were obtained from the Cell Bank/Stem Cell Bank of the Chinese Academy of Sciences (Shanghai, China). All cell lines were maintained in Dulbecco's Modified Eagle Medium (Gibco, USA) supplemented with 10% FBS and 1% penicillin-streptomycin in a humidified incubator containing 5% CO_2_ at 37°C.

**Colony formation assay**

Cells were seeded into 6-well plates at a density of 1 × 10³ cells per well. After 14 days of incubation, the colonies were fixed with 4% paraformaldehyde and stained with 0.5% crystal violet to visualize the colony area. Cell confluence in each well was quantified using ImageJ software.

**Plasmids and siRNA transfection**

Plasmids and siRNAs were designed and synthesized by GenePharma (Shanghai, China). For transient transfection, cells were seeded into 6-well plates and grown to 60%70% confluence before being transfected with plasmids or siRNA using Lipofectamine 3000 (Invitrogen, USA) following the manufacturer’s instructions.

The chromatographic column was equilibrated with 95% Buffer A prior to sample injection and separated through an analytical column. After chromatographic separation, peptide samples were subjected to mass spectrometry analysis using a high-resolution mass spectrometer in positive ion detection mode.

**Immunofluorescence**

For immunofluorescence assays, cells and tissues were fixed in 4% paraformaldehyde solution for 30 min and permeabilized with 0.2% Triton X-100 at room temperature. Samples were then blocked with 5% bovine serum albumin for 1 h and incubated with the indicated primary antibodies overnight at 4°C. After washing, the samples were incubated with secondary antibodies. Fluorescent images were captured using a fluorescence microscope (Leica Microsystems, Germany).

***In vivo* biodistribution and tumor targeting of NPs**

To assess the *in vivo* biodistribution and tumor-targeting ability of the NPs, free-RhB and RhB-NPs were injected via the tail vein. Dynamic fluorescence imaging was performed at the indicated time points using a small-animal live imaging system (AniView 100, China). The animal experiments were repeated, and organs along with tumors were harvested 48 h post-injection for *ex vivo* fluorescence imaging.

***In vitro* drug release behavior**

The dynamic dialysis method was used to evaluate the *in vitro* drug release profile of si-HECTD2#3 NPs. The si-HECTD2#3 NPs suspension was placed into a dialysis bag, which was immersed in a bottle filled with PBS containing Tween 80. At the indicated time points, aliquots of the release medium were collected and replaced with fresh PBS. The concentration of released si-HECTD2 was determined using a standard curve for quantification.

**ChIP-qPCR assay**

To evaluate the enrichment of H3K18la in the promoter region of HECTD2, ChIP-qPCR assays were conducted using the Pierce Agarose ChIP Kit (Thermo Fisher Scientific, USA) following the manufacturer’s instructions. Following ultrasonic fragmentation, protein-DNA complexes were immunoprecipitated using a primary antibody specific to H3K18la or an IgG control.

Additional file 2: Supplementary figures


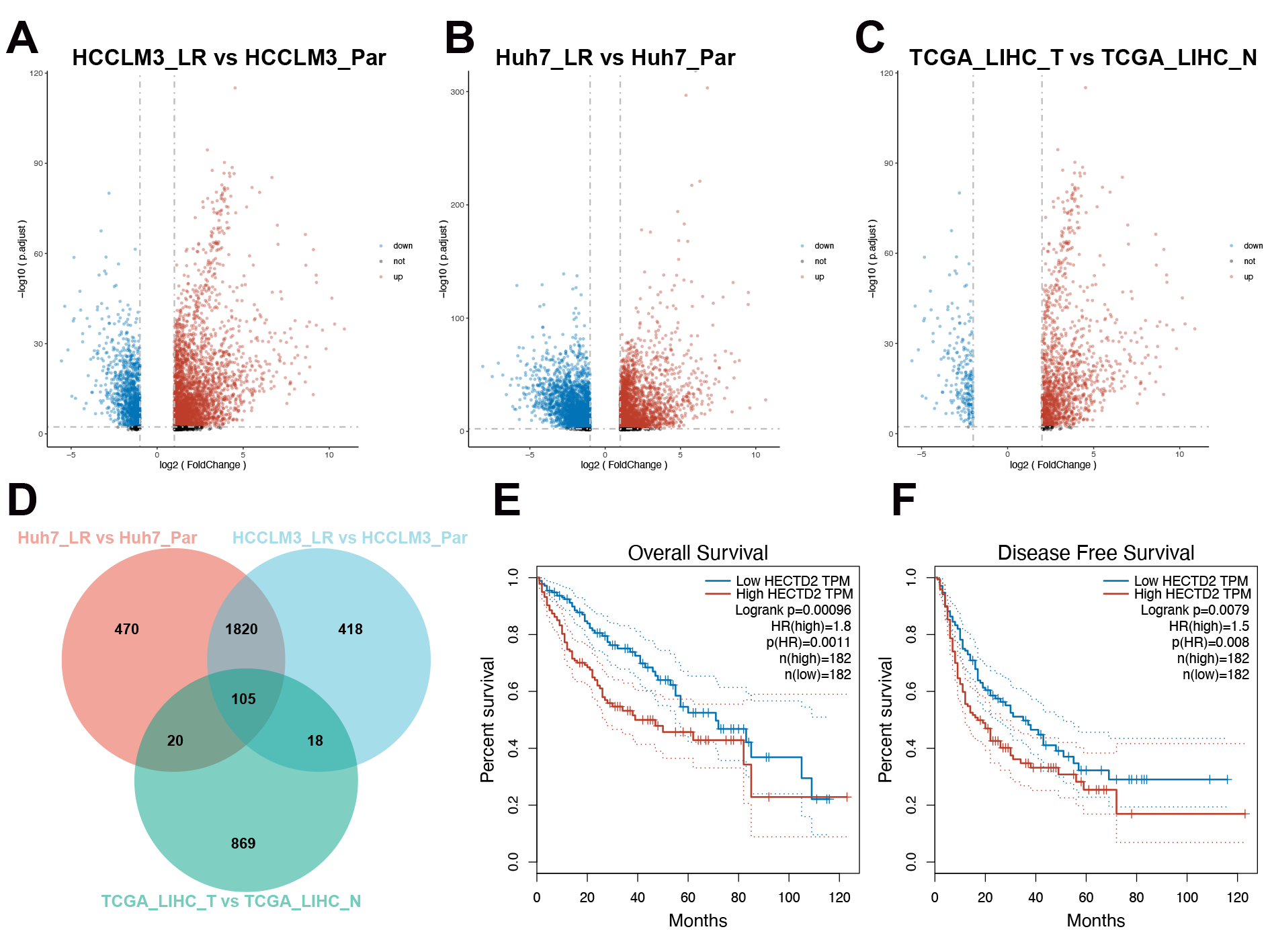


**Figure S1** HECTD2 is associated with lenvatinib resistance and predicted unfavorable survival outcomes in HCC. A) Volcano plot of the differentially expressed proteins between lenvatinib-resistant HCCLM3-LR and parental HCCLM3 cells. B) Volcano plot of the differentially expressed proteins between lenvatinib-resistant Huh7-LR and parental Huh7 cells. C) Volcano plot of the differentially expressed molecules between HCC tumors and nontumor samples in TCGA-LIHC database. D) Venn diagram representing the intersection of the three sets. E) Kaplan-Meier curves showing the disparity in overall survival between HECTD2 high expression group and HECTD2 low expression group. F) Kaplan-Meier curves showing the disparity in disease-free survival between HECTD2 high expression group and HECTD2 low expression group.
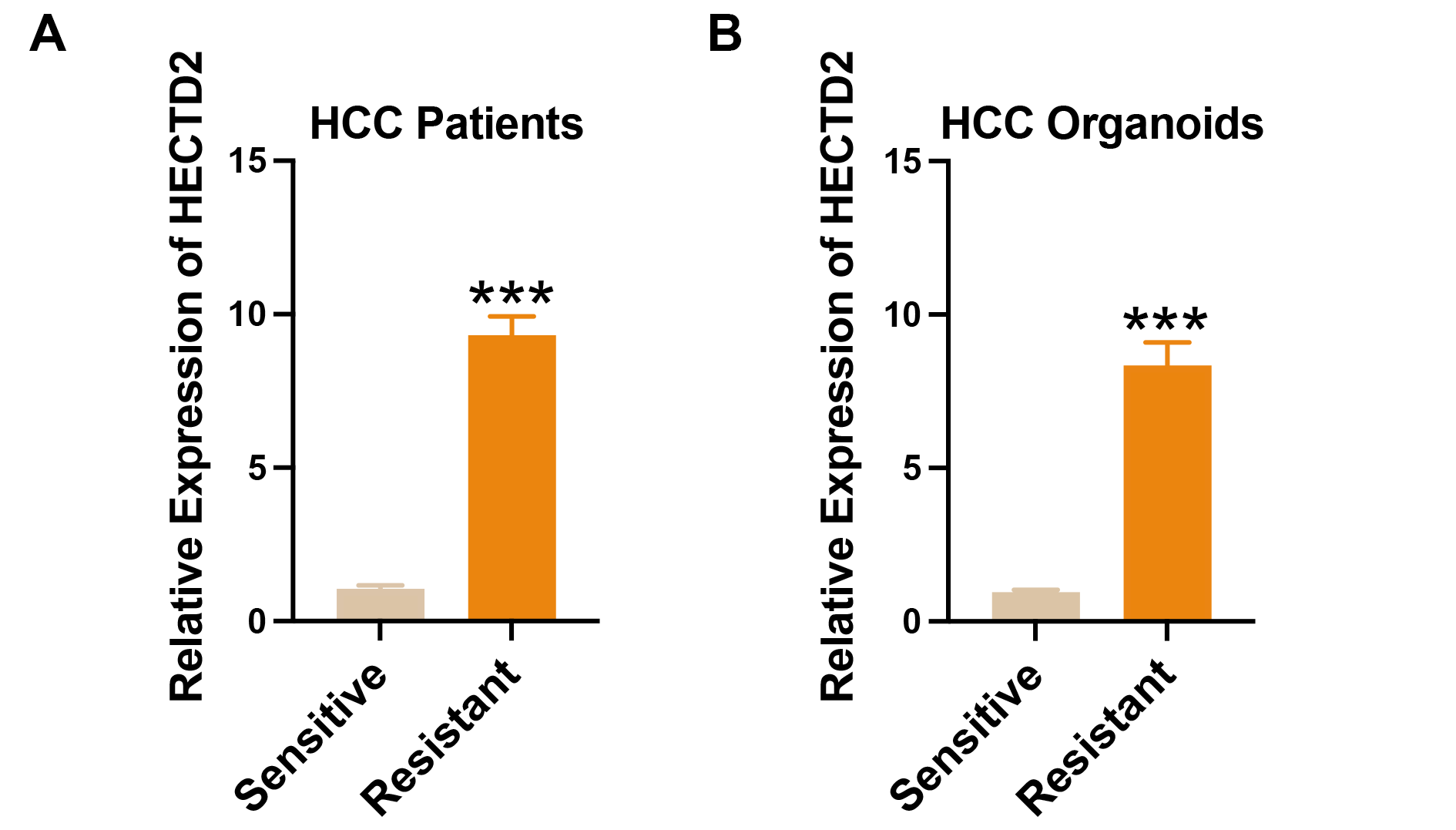


**Figure S2** HECTD2 mRNA expression level is elevated in lenvatinib-resistant organoids and the original patient tumor samples. A) HECTD2 mRNA expression level in the original lenvatinib-sensitive and lenvatinib-resistant patient tumor samples. B) HECTD2 mRNA expression level in lenvatinib-sensitive and lenvatinib-resistant organoids. ****P* < 0.001.


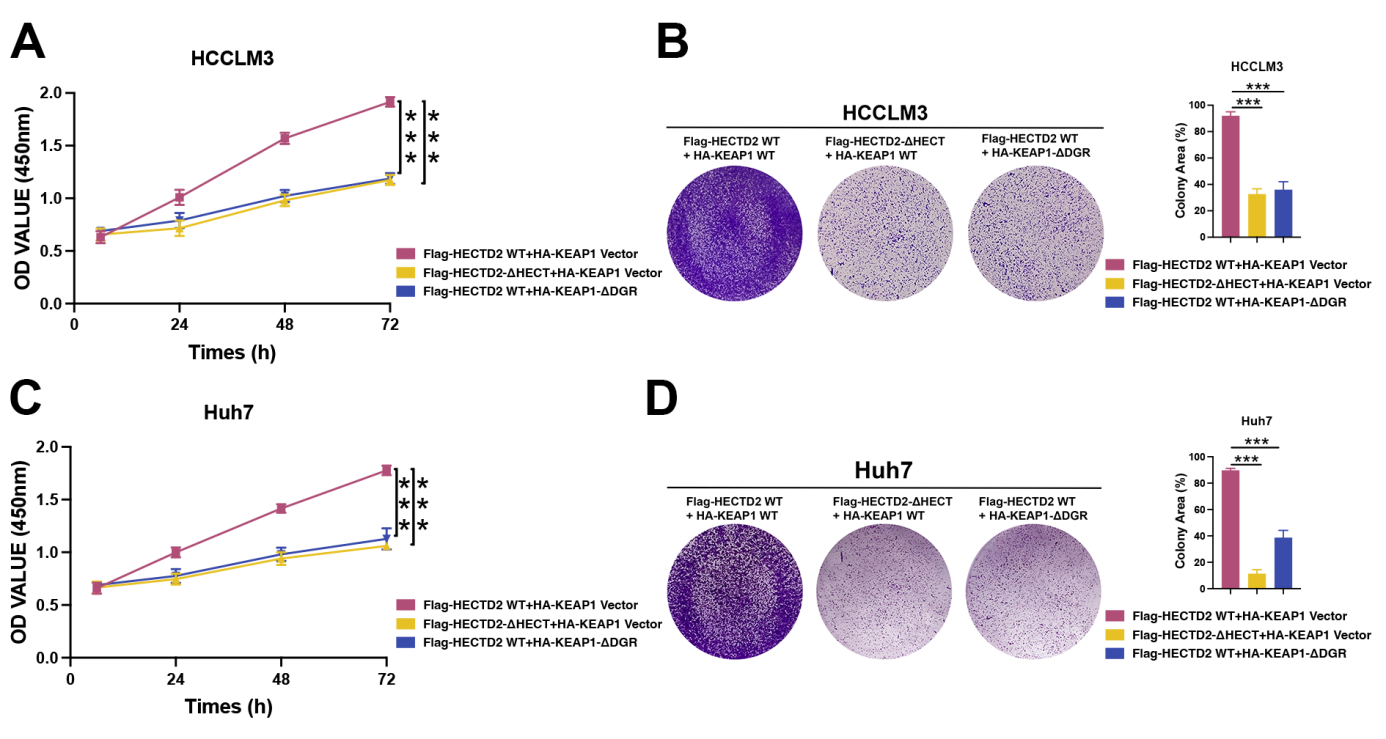


**Figure S3** The DGR domain of KEAP1 and the HECT domain of HECTD2 mediated their interaction. A) CCK-8 assay showing the effects of KEAP1-ΔDGR and HECTD2-ΔHECT on cell proliferation in HCCLM3 cells. B) Colony formation assay showing the effects of KEAP1-ΔDGR and HECTD2-ΔHECT on the proliferative capability of HCCLM3 cells. C) CCK-8 assay showing the effects of KEAP1-ΔDGR and HECTD2-ΔHECT on cell proliferation in Huh7 cells. D) Colony formation assay showing the effects of KEAP1-ΔDGR and HECTD2-ΔHECT on the proliferative capability of Huh7 cells. ****P* < 0.001.


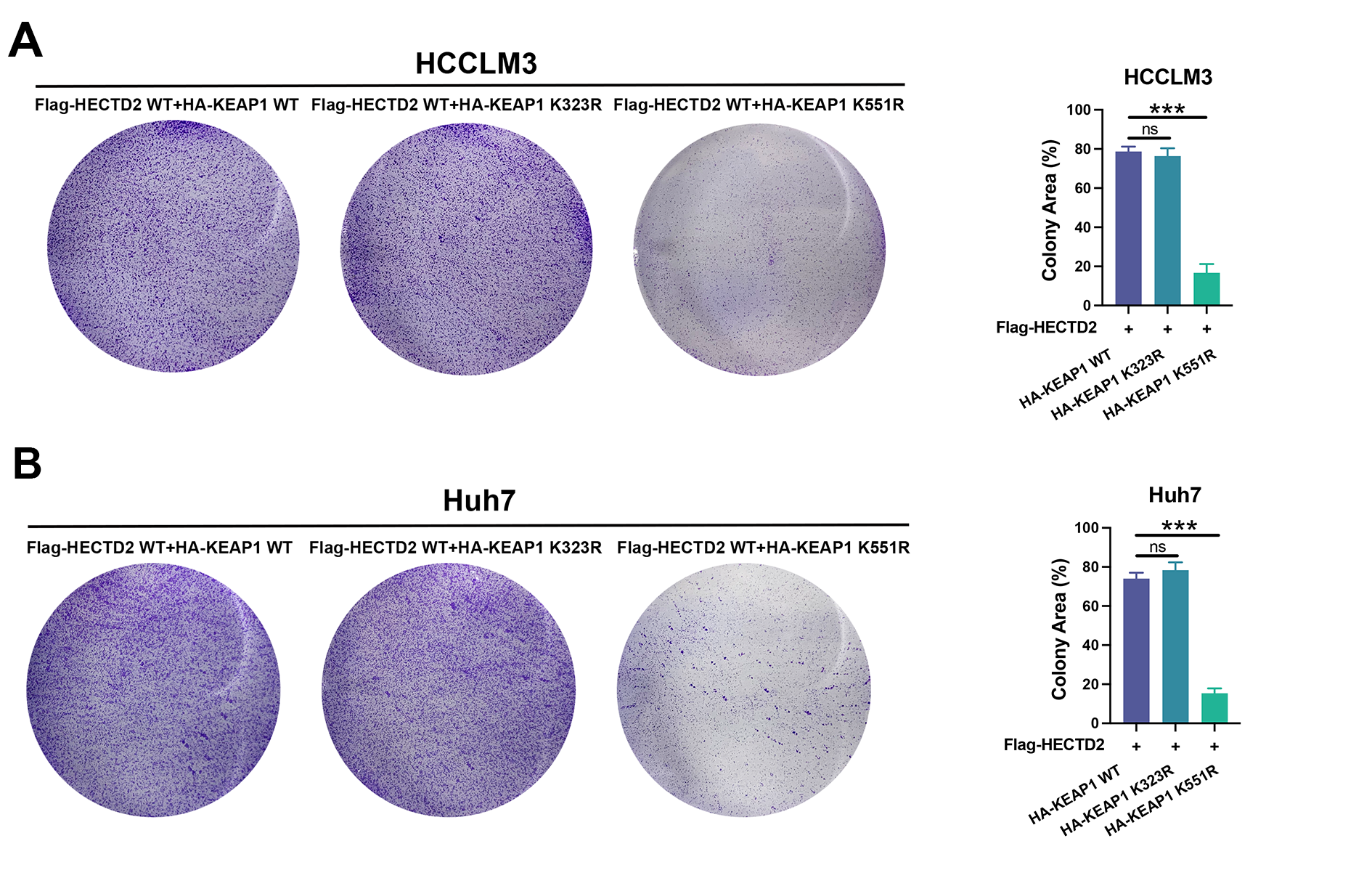


**Figure S4**  KEAP1 is ubiquitinated at the K551 site as a substrate for HECTD2. A) Colony formation assay showing the effects of KEAP1 mutant plasmids on the proliferative capability of HCCLM3 cells. B) Colony formation assay showing the effects of KEAP1 mutant plasmids on the proliferative capability of Huh7 cells.


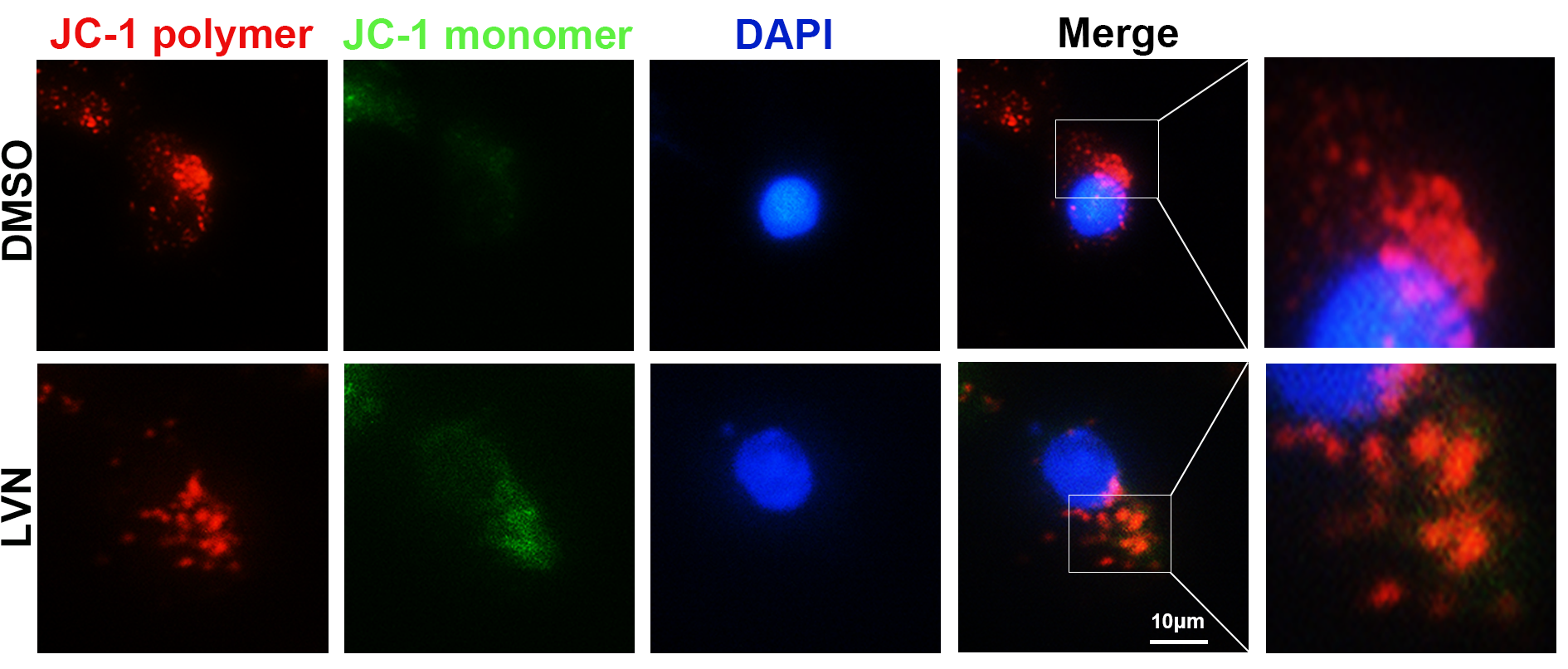


**Figure S5** Immunofluorescence showing JC-1 polymer and JC-1 monomer in HCC cells exposed to lenvatinib (LVN) treatment.


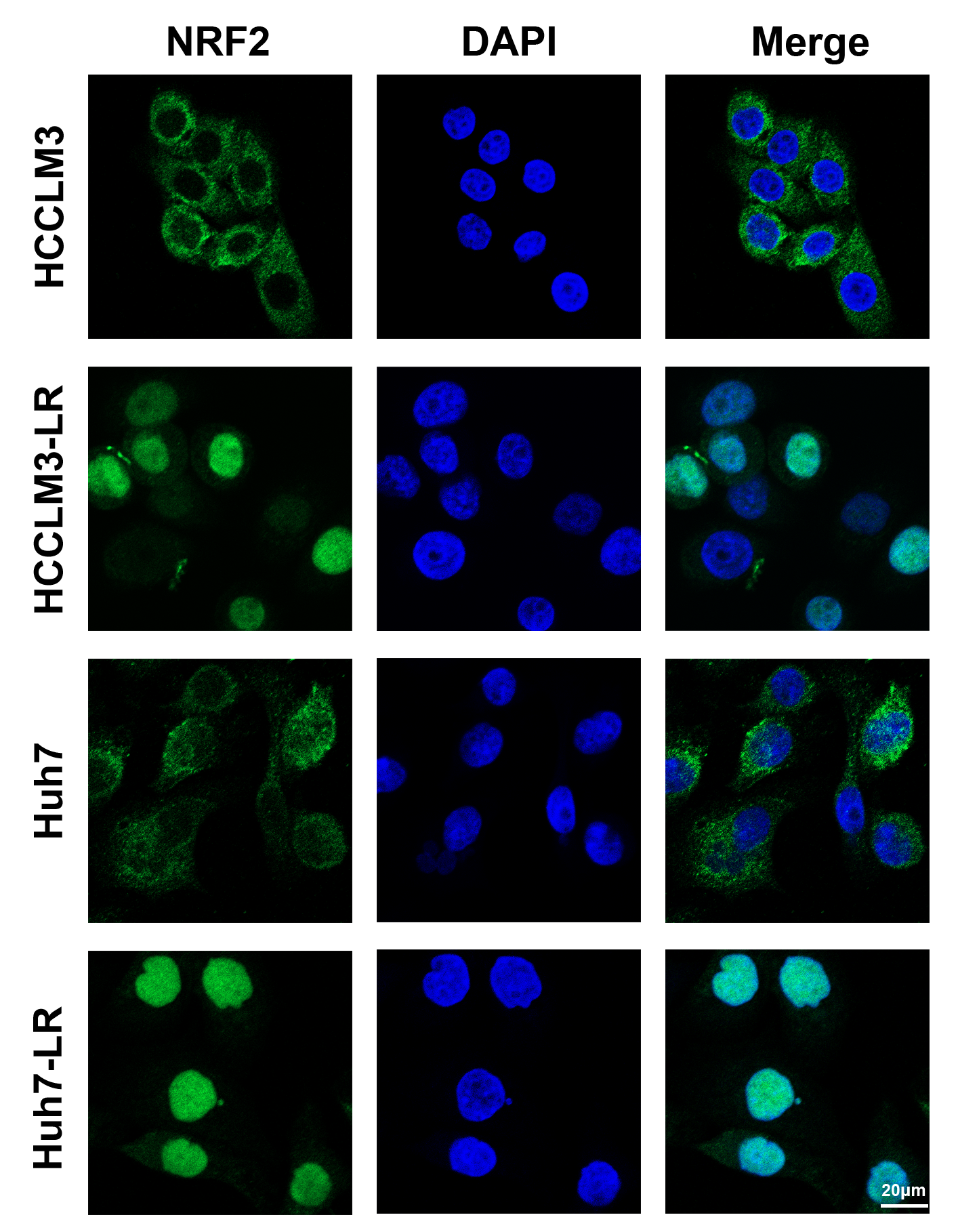


**Figure S6** The expression of NRF2 in the cytoplasm and nucleus.
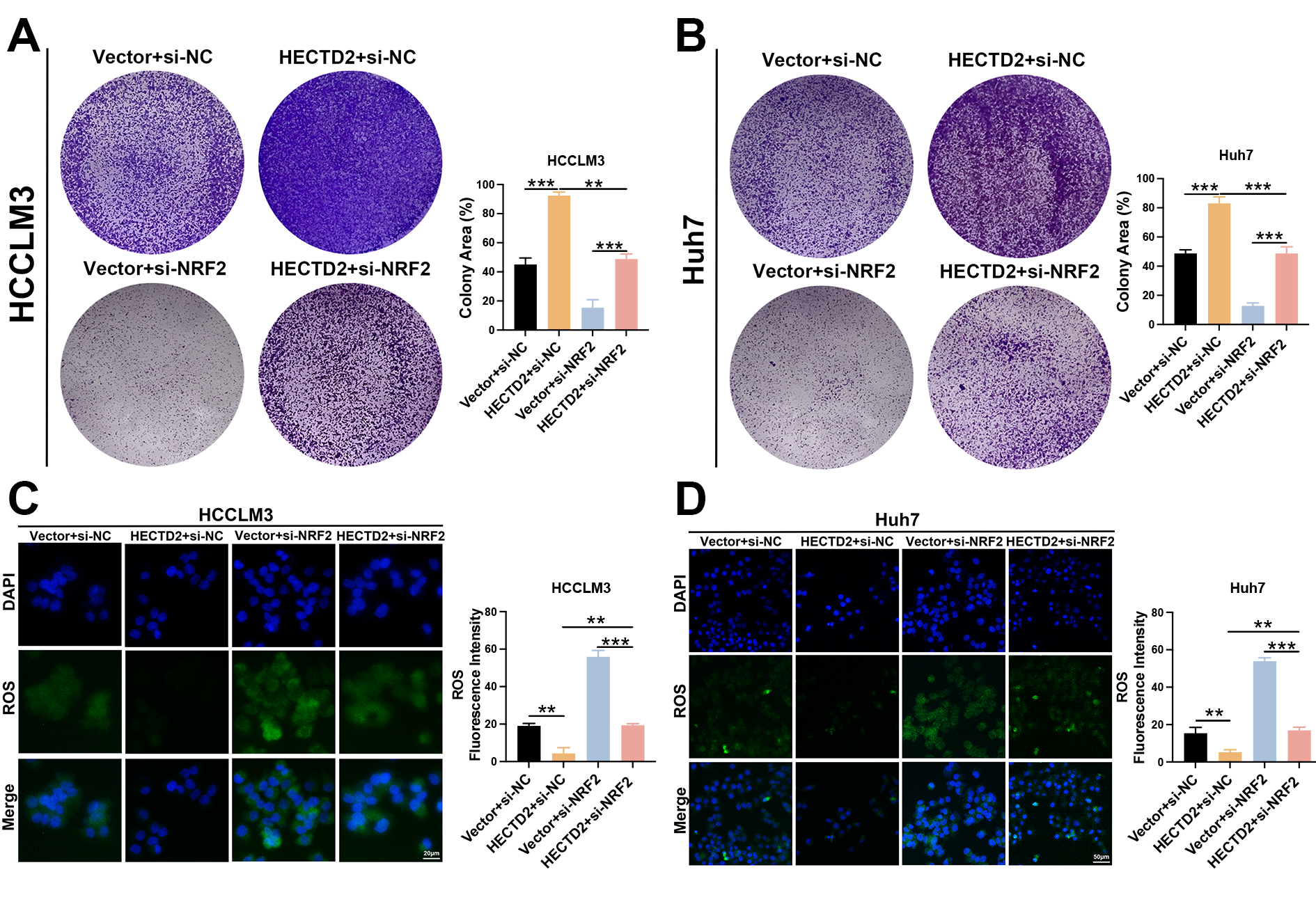


**Figure S7** NRF2 knockdown counteracts the elevated cell growth and decreased ROS in HECTD2-overexpressing cells. A) Colony formation assay showing the effects of NRF2 knockdown on the proliferative capability of HECTD2-overexpressing HCCLM3 cells. B) Colony formation assay showing the effects of NRF2 knockdown on the proliferative capability of HECTD2-overexpressing Huh7 cells. C) The effects of NRF2 knockdown on ROS in HECTD2-overexpressing HCCLM3 cells. D) The effects of NRF2 knockdown on ROS in HECTD2-overexpressing Huh7 cells. ***P* < 0.01; ****P* < 0.001.


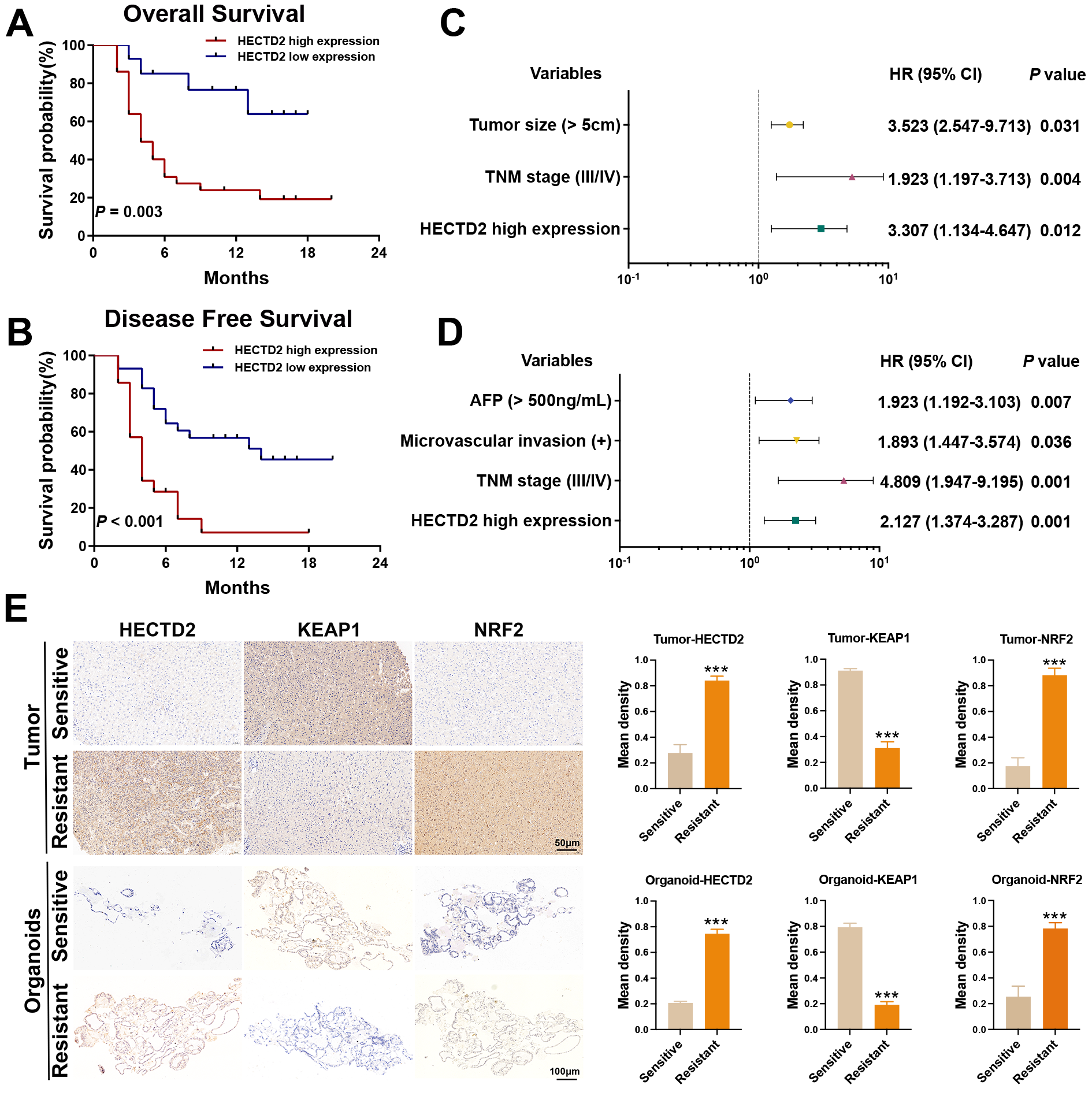


**Figure S8** HECTD2 level is clinically relevant to HCC prognosis and lenvatinib resistance. A) Kaplan-Meier survival analysis showing overall survival of HECTD2^high^ and HECTD2^low^ HCC patients. B) Kaplan-Meier survival analysis showing disease-free survival of HECTD2^high^ and HECTD2^low^ HCC patients. C) Cox multivariate regression analyses showing the independent risk factors for OS. D) Cox multivariate regression analyses showing the independent risk factors for DFS. E) IHC staining of HECTD2, KEAP1, and NRF2 in lenvatinib-resistant and lenvatinib-sensitive HCC organoids and the original tumor tissues. ****P* < 0.001.


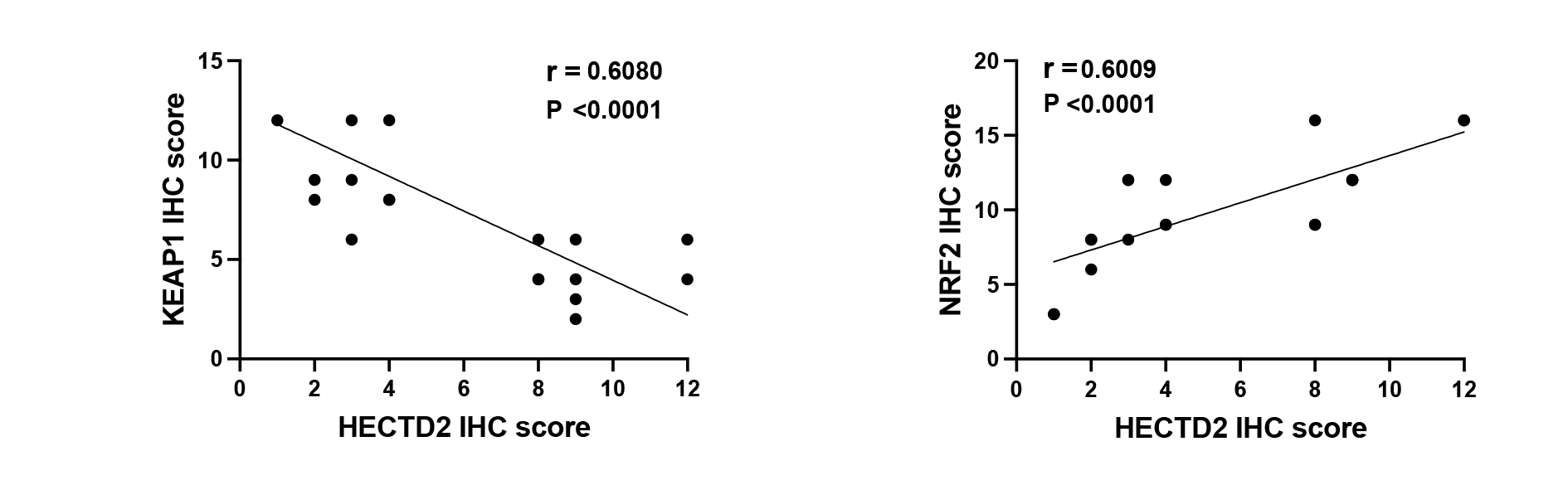


**Figure S9** The expression correlation of HECTD2 with NRF2 and KEAP1 in HCC samples.
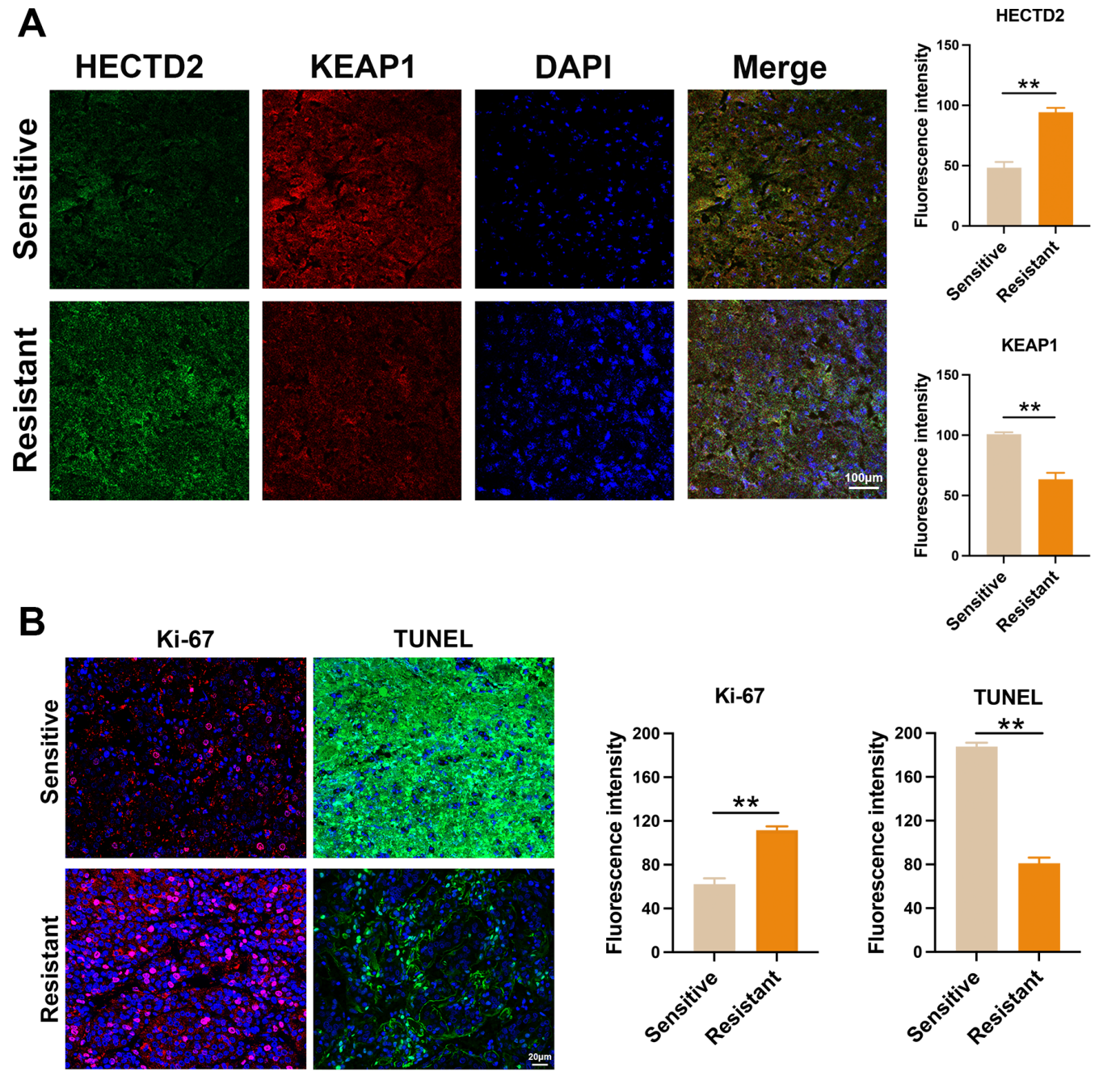


**Figure S10** HECTD2 level is clinically relevant to lenvatinib resistance in HCC. A) Immunofluorescence demonstrating HECTD2 and KEAP1 expression in lenvatinib-sensitive and lenvatinib-resistant tumor tissues. B) Ki-67 level and TUNEL staining in lenvatinib-sensitive and lenvatinib-resistant tumor tissues. ***P* < 0.01.


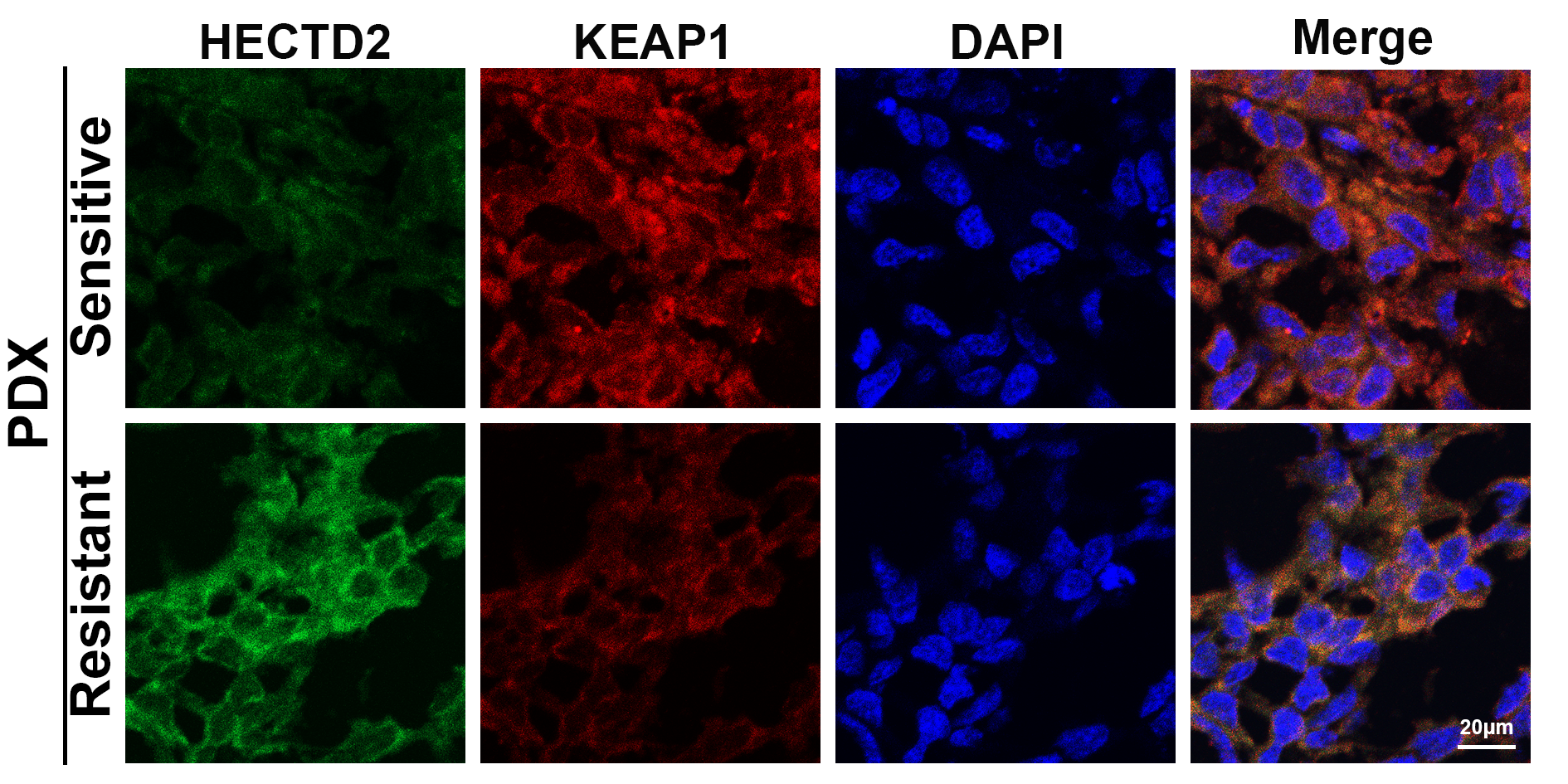


**Figure S11** Immunofluorescence results of HECTD2 and KEAP1 in xenografts derived from lenvatinib-sensitive and lenvatinib-resistant PDX models.


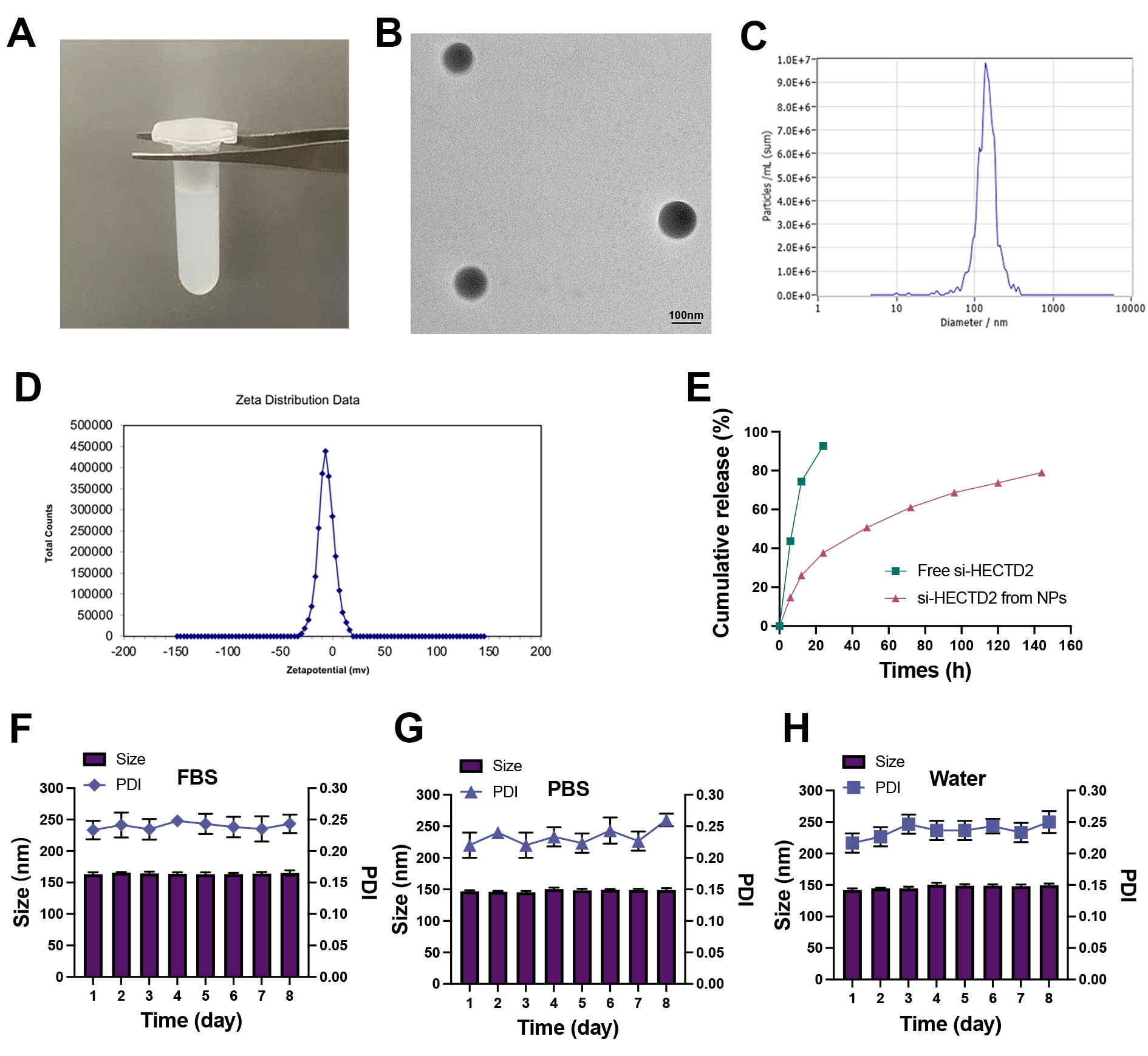


**Figure S12** The preparation and characterization of PLGA-PEG(si-HECTD2#3) NPs. A) PLGA-PEG(si-HECTD2#3) NPs was milky white in solution. B) Morphology of PLGA-PEG(si-HECTD2#3) NPs by transmission electron microscopy (TEM). C) Particle size of the NPs. D) Zeta potential of the NPs. E) The releasing behavior of si-HECTD2#3 from PLGA-PEG(si-HECTD2#3) NPs. F) The particle size and PDI of the NPs in FBS. G) The particle size and PDI of the NPs in PBS. H) The particle size and PDI of the NPs in water.


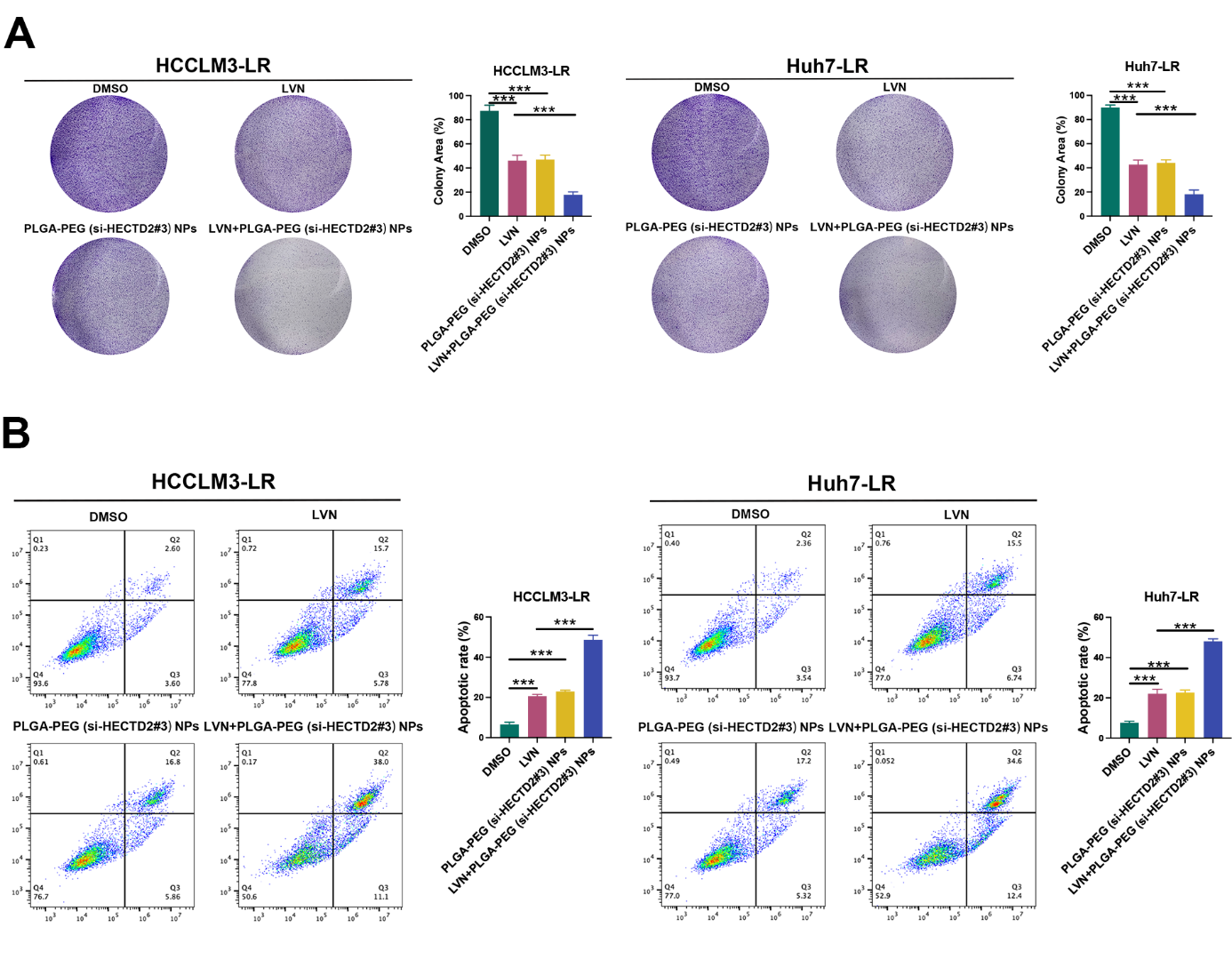


**Figure S13** The antitumor efficiency of the successfully constructed PLGA-PEG(si-HECTD2#3) NPs. A) Apoptosis assay showing the therapeutic effect of PLGA-PEG(si-HECTD2#3) NPs in lenvatinib-sensitive cells. B) Colony formation assay showing the therapeutic effect of PLGA-PEG(si-HECTD2#3) NPs in lenvatinib-sensitive cells.
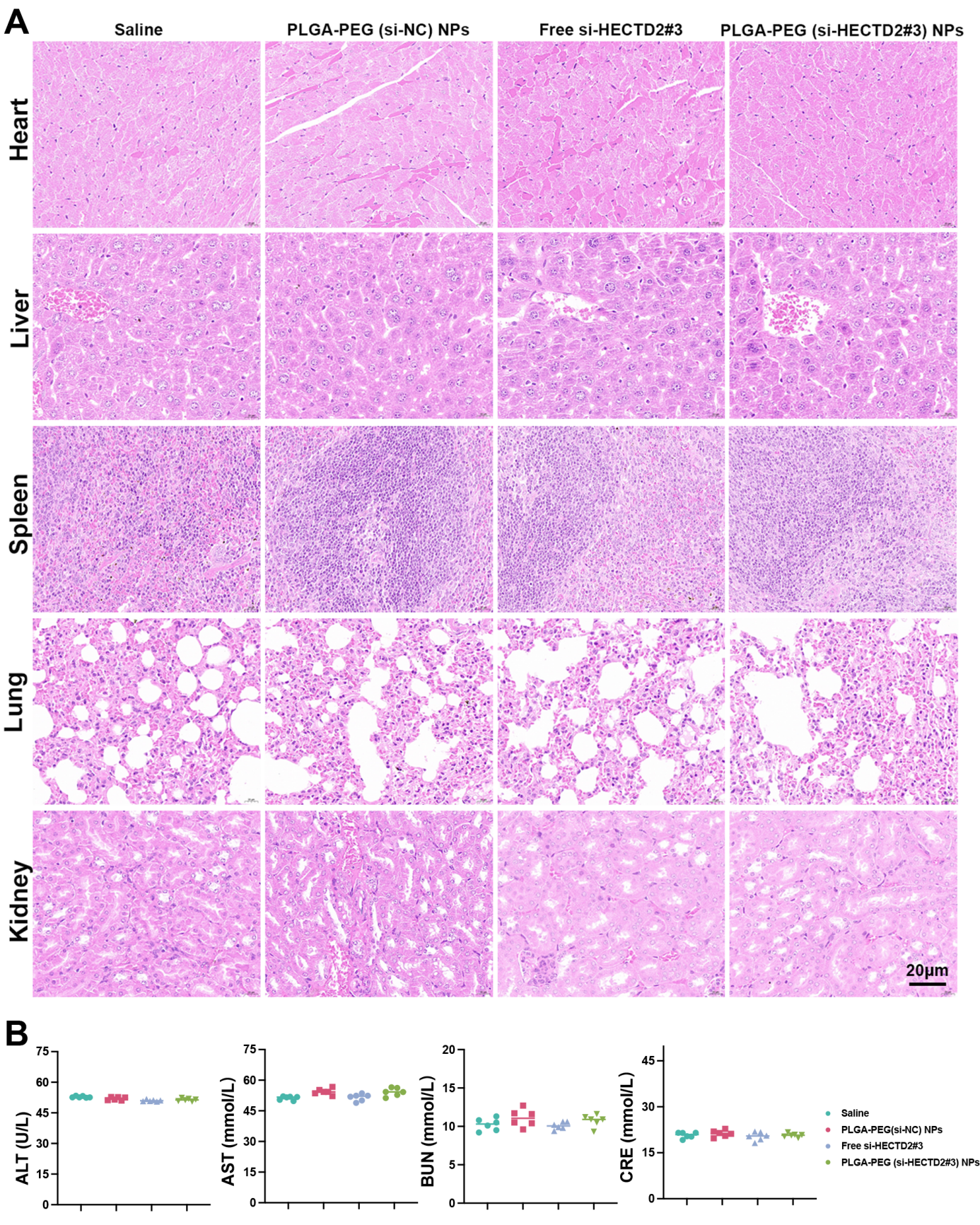


**Figure S14** PLGA-PEG(si-HECTD2#3) NPs cause no significant toxicity to major organs. A) H&E results of the heart, liver, spleen, lung, and kidney. B) Serum levels of alanine aminotransferase (ALT), aspartate aminotransferase (AST), creatinine (CRE), and blood urea nitrogen (BUN).


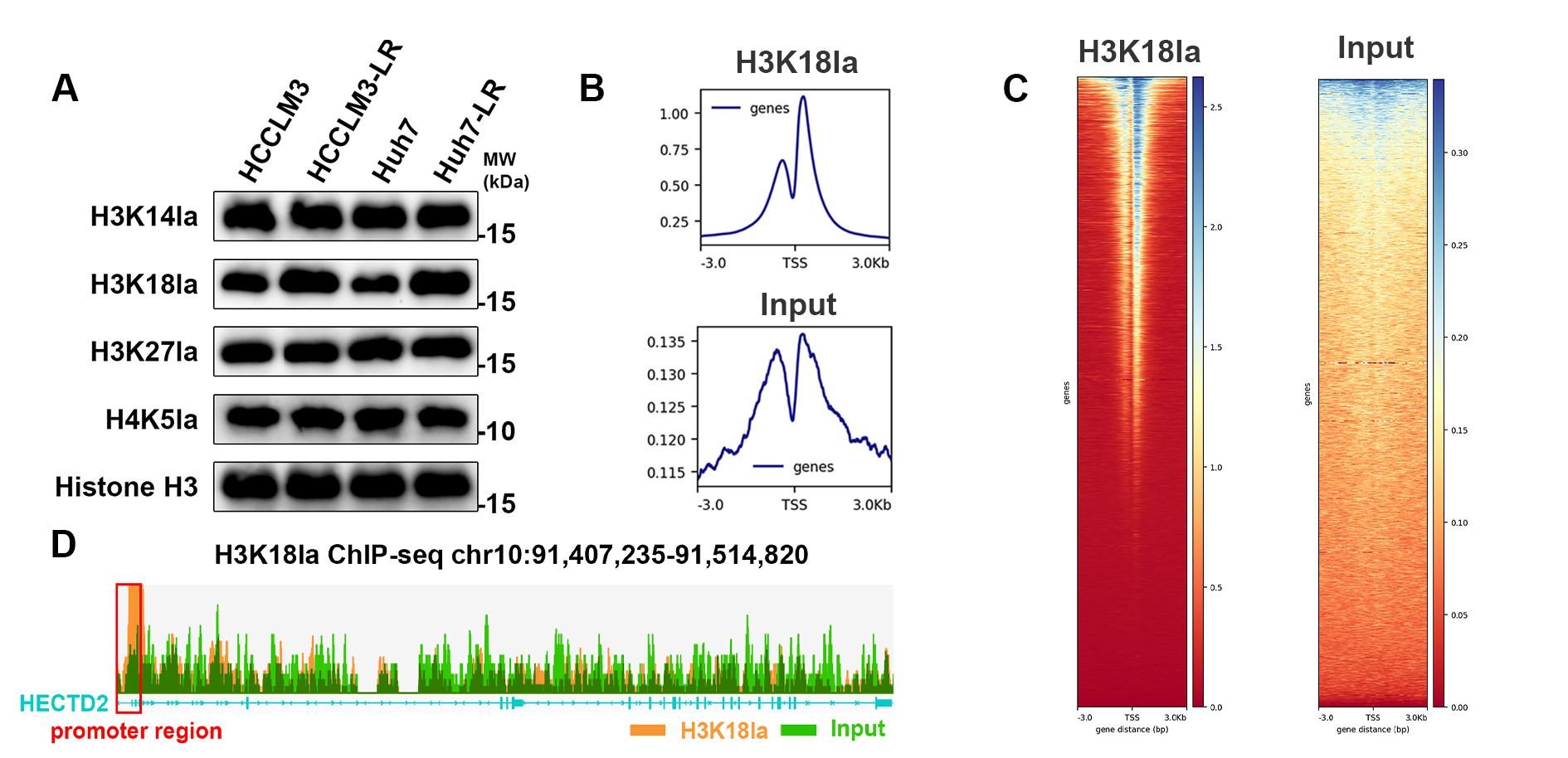


**Figure S15** H3K18la drives HECTD2 transcription. A) The levels of H3K14la, H3K18la, H3K27la, and H4K5la in lenvatinib-sensitive and lenvatinib-resistant HCC cells. B) ChIP-seq analysis of H3K18la genomic binding at target sites. A 3-kb interval centered on each H3K18la peak is demonstrated. C) Heatmap showing the binding sites of H3K18la by ChIP-seq. D) ChIP-seq graph of HECTD2 promoter bound by H3K18la.
